# Ligand induced dissociation of the AR homodimer precedes AR monomer translocation to the nucleus

**DOI:** 10.1038/s41598-019-53139-9

**Published:** 2019-11-13

**Authors:** Ryota Shizu, Kosuke Yokobori, Lalith Perera, Lee Pedersen, Masahiko Negishi

**Affiliations:** 1 Pharmacogenetic section, Reproductive and Developmental Biology Laboratory, National Institute of Environmental Health Sciences, National Institutes of Health, Research Triangle Park, North Carolina, 27709 USA; 2 Genome Integrity and Structural Biology Laboratory, National Institute of Environmental Health Sciences, National Institutes of Health, Research Triangle Park, North Carolina, 27709 USA

**Keywords:** Steroid hormones, Transcriptional regulatory elements

## Abstract

The androgen receptor (AR) regulates male sexual development. We have now investigated AR homodimerization, hormone-dependent monomerization and nuclear translocation in PC-3 and COS-1 cells, by utilizing mutations associated with the androgen insensitivity syndrome: Pro767Ala, Phe765Leu, Met743Val and Trp742Arg. AR wild type (WT) was expressed as a homodimer in the cytoplasm, while none of these mutants formed homodimers. Unlike AR WT which responded to 1 nM dihydrotestosterone (DHT) to dissociate and translocate into the nucleus, AR Pro767Ala and Phe765Leu mutants remain as the monomer in the cytoplasm. In the crystal structure of the AR LBD homodimer, Pro767 and Phe765 reside closely on a loop that constitutes the dimer interface; their sidechains interact with the Pro767 of the other monomer and with the DHT molecule in the ligand-binding pocket. These observations place Phe765 at a position to facilitate DHT binding to Pro767 and lead to dissociation of the AR homodimer in the cytoplasm. This Pro-Phe Met relay may constitute a structural switch that mediates androgen signaling and is conserved in other steroid hormone receptors.

## Introduction

The androgen receptor (AR), a member of the nuclear steroid hormone receptor (NR3C) subfamily, regulates male sexual development^1^. Androgen directly binds AR in the cytoplasm and translocates it into the nucleus, conferring its physiology by activating various genes^2,3^.

AR is believed to form a homodimer for binding and activating its target genes. Recently, an X-ray crystal structure of AR LBD homodimer was determined, in which three loops constitute the dimer interface (hereafter call it D3L)^4^. Polymorphic mutations associated with the androgen insensitivity syndrome (AIS) are observed in this D3L interface of AR molecule^4^. However, it remains elusive as to how these mutations affect androgen sensitivities of AR through its homodimerization.

Nuclear non-steroid hormone receptors heterodimerize with retinoid X receptor (RXR) through a well-established interface that includes helices 10 and 11 in their X-ray crystal structures^5–8^, (hereafter called the H10/11 interface). Similarly, estrogen receptor α (ERα) and hepatocyte nuclear factor 4α (HNF4α) are also reported to form their homodimers through this H10/11 interface^9,10^. The D3L interface was first observed in the X-ray crystal structure of the glucocorticoid receptor (GR) homodimer^11^. The D3L interface resides on an area of opposite surface of the H10/11 interface of the AR molecule^9,10^. However, a recent confirmation that the AR LBD utilizes the D3L interface to homodimerize strengthens the notion that this interface may be physiologically relevant^4^. In addition, the nuclear receptor CAR (NR1I3) has been found to form a homodimer through its D3L interface in the cytoplasm which dissociates upon activation and translocation into the nucleus^12,13^. To advance the biology of the D3L interface, we have now investigated whether (how) AIS-related mutations affect AR homodimerization and nuclear translocation in cells.

The AIS-associated mutations, Pro767Ala^14^, Phe765Leu^15^, Met743Val^16^ and Trp742Arg^15^ were further examined by employing various experimental methods including site-directed mutagenesis, co-immunoprecipitation of GFP- and FLAG-tagged proteins ectopically-expressed in cells, 2-dementional electrophoresis, cell-based reporter assays and confocal analysis of GFP tagged proteins in cells. Pro625 of GR corresponds to Pro767 of AR was previously investigated as a critical residue in GR homodimerization^11^. In this study, we demonstrated that AR WT formed a homodimer that dissociated and nuclear translocated in response to dihydrotestosterone (DHT) in COS-1 cells. With this background, AR mutants (Pro767Ala, Phe765Leu, Met743Val and Trp742Arg) were expressed in COS-1 cells to examine their roles in AR homodimerization. In addition, these residues are mapped to AR molecules based on its X-ray crystal structure to understand structure-function relationships. This mapping was then extended to other steroid hormone receptors. Here we present experimental observations in support of the hypothesis that the AR homodimer responds to androgen in the cytoplasm by dissociation and nuclear translocation, and, additionally, that an intramolecular Pro-Phe-Met relay regulates the androgen signal.

## Methods

### Regents and materials

Dexamethasone (DEX), 5α-dihydrotestosteron (DHT), R1881 and HRP-conjugated anti-FLAG M2 were purchased from Sigma-Aldrich (St. Louis, MO). HRP conjugated antibodies against rabbit IgG and SP-1 (sc-059) and histone H1 (FL-219) antibodies were purchased from Santa Cruz Biotechnology (Dallas, TX). The antibody against HSP90 was purchased from Cell Signaling (Danvers, MA). The antibody against GFP (HRP-conjugated) was purchased from Abcam (Cambridge, MA). An enhanced chemiluminescence reagent WesternBrighTM was from GE Healthcare (Piscataway, NJ). The α−tubulin antibody (21445) was obtained from Cell Signaling Technology. Anti-GFP agarose beads for immunoprecipitation was provided by the Protein Expression Core Facility in NIEHS^13^.

### Plasmids

Human AR cDNA (NM_000044.6) was cloned into pEGFP-c1, pECFP-c1, pEYFP-c1 (Clontech Laboratories, Palo Alto, CA) and pcDNA3.1 (Invitrogen, Carlsbad, CA). A DNA fragment of FLAG tag was placed at the 5′ end of AR in pcDNA3.1. human *PSA* promoter (region -430 to +12) were inserted into XhoI and BglII sites of pGL3 plasmid (Promega). 3xGRE-TK-pGL3, GR-pCR3.1, pEGFP-GR and pECFP-GR were constructed previously^17^. Using a PrimeSTAR Max DNA Polymerase (Clontech) and appropriately mutated primers, we constructed all the mutants and confirmed them by nucleotide sequencing. Plasmids were transfected into cultured cells with Lipofectamine2000 (Invitrogen) according to the manufacturer’s instructions.

### Cell culture and fractionation

Cells were cultured on 100-mm culture dishes to about 70% confluency with serum free DMEM high glucose medium with or without 10 nM DHT for 24 hr. Whole cell extracts were prepared by centrifuging cell homogenates in lysis buffer containing Tris-HCl (pH 8.0), 0.5 mM EDTA, 100 mM NaCl, 1% Triton X-100 and 10% glycerol. For fractionation, cells were rinsed with PBS and suspended in 1 mL of cytoplasm extraction buffer (10 mM HEPES (pH 7.5), 10 mM KCl, 1.5 mM MgCl_2_). The cell suspensions were centrifuged at 2000x g for 5 min. Resulted supernatants were collected as crude cytosolic fraction which was centrifuged at 20000x g for 30 min and its supernatant was collected as cytosolic fraction. Cell pellets were resuspended in 1 mL of wash buffer (10 mM HEPES (pH 7.5), 10 mM KCl, 1.5 mM MgCl_2_, 0.075% NP-40), centrifuged at 2000 × g for 5 min. Resulted pellets were resuspended in 400 μL of nucleus extraction buffer (10 mM HEPES (pH 7.5), 100 mM KCl, 3 mM MgCl_2_, 0.1 mM EDTA), incubated for 30 min on ice and centrifuged at 20000 × g for 30 min, supernatants from which the nucleus fraction was collected. A portion of nuclear fraction was resuspended in 400 μL of DNA-binding protein extraction buffer (10 mM HEPES (pH 7.5), 400 mM NaCl, 100 mM KCl, 3 mM MgCl_2_, 0.1 mM EDTA), incubated for 30 min on ice and centrifuged at 20000 × g for 30 min to obtain the DNA-binding protein fraction.

### Co-immunoprecipitation

Twenty-four hours after seeding, COS-1 cells were co-transfected with expression plasmids for GFP- and FLAG-tagged ARs and cultured in serum free DMEM media for additional 24 hours. These cells were treated with 0.1%DMSO or DHT for 1 hour in serum free media. Then, cells were lysed with the lysis buffer containing Tris-HCl (pH 8.0), 0.5 mM EDTA, 100 mM NaCl, 1% Triton X-100 and 10% glycerol. Cell lysate was incubated with an anti-GFP agarose at 4 °C for 12 h and, subsequently, washed with lysis buffer for three times, and eluted with SDS-PAGE sample buffer and subjected to Western blot analysis. In addition, FLAG-ARs and GFP were co-expressed as a control group. We confirmed that FLAG-ARs wasn’t precipitated by GFP co-expressed samples in all experiments.

### Western blot

Western blot samples were subjected to SDS-PAGE on 10% polyacrylamide gels following transblot to an Immobilon-P Transfer Membrane (Millipore Corporation, Billerica, MA). Membranes were blocked for 1 h with 5% non-fat dried milk in 0.05% Tween 20 containing Tris-buffered saline (TBST). The membranes were incubated for 16 h with the 1000-fold diluted primary antibody in TBST and incubated for 1 h with 10000-fold diluted HRP-conjugated secondary antibody. After incubating with secondary antibody, proteins were detected by an enhanced chemiluminescence reagent Western Bright ECL (GE Healthcare). PageRuler Plus Prestained Protein Ladder (Thermo Fisher Scientific) was run as a molecular weight marker. Uncropped images of blots are shown in Supplemental Figs 5–8. Quantifications of band intensities were performed by using ImageJ software (NIH) by following the user guide and are shown in Supplemental Figs 5–8.

### Two-dimensional gel electrophoresis

Blue native/SDS-PAGE was performed as previously described^12^. Cells were homogenized in buffer containing 20 mM HEPES (pH 7.6), 150 mM NaCl, 15% Glycerol, 1% Triton X-100 and 1 mM EDTA. Cell lysates were subjected to electrophoresis on a 4–16% gradient Native gel (Invitrogen). The gel was placed onto 10% SDS-PAGE gel and western blot analysis was carried out.

### Reporter assay

PC-3 cells were transfected with reporter plasmid (pGL3), phRL-TK control plasmid (Promega) and pcDNA3.1 expression plasmid for ARs or GRs with Lipofectamine 2000 and treated with ligands in the serum free media for 24 h. Cell lysate was subjected to Dual Luciferase Assay System (Promega). Firefly luciferase activity was normalized to Renilla luciferase activity.

### Cytochemistry

PC-3 or Huh-7 cells (1 × 10^5^ cells/well) were grown on 35 mm glass coverslips dishes (Corning, NY) and transfected with a given expression plasmid for GFP, CFP or YFP-labeled proteins. Twenty-four hours after transfection, media was changed to serum free DMEM media and cells were treated with DEX or DHT for 1 h. Cells were fixed with methanol and fluorescence was visualized using a Zeiss LSM 710 confocal microscope (Zeiss, Oberkochen, Germany)

### Molecular dynamic simulations

Using molecular dynamics (MD), solution structures of the androgen receptor, its dimer form, and several mutants were generated. The initial structure of androgen receptor for simulations was taken from the homodimeric X-ray crystal structure from the PDB code 5JJM. Desired mutations (W742R, M743V, F765L, P767A) were introduced using the program Coot^18^. Simulations were carried out with and without the ligand, DHT. Each system was solvated in a box of water and each box was selected so that box boundaries were at least 20 A from the closest protein atom. Prior to equilibration, all systems were subjected to (1) 100-ps belly dynamics runs with fixed peptide, (2) minimization, (3) low temperature constant pressure dynamics at fixed protein to assure a reasonable starting density, (4) minimization, (5) step-wise slow heating molecular dynamics at constant volume, and (6) constant volume unconstrained molecular dynamics for 10 ns. All final unconstrained trajectories were calculated at 300 K under constant pressure (for 310 ns, time step 1 fs) using the PMEMD module of Amber.18 to accommodate long range interactions^19^. The parameters were taken from the FF14SB force field for proteins and the DHT parameters were from the gaff2 force field in the AMBER.18 package. Partial charges on DHT were calculated using Gaussian.09^20^ at the B3LYP/6–31 g level. Ligand binding free energies were calculated with the help of the MMPBSA module of AMBER.18 using default parameters and equally spaced 200 configurations selected from the last 200 ns of the molecular dynamics trajectory. MD trajectories of dimers (WT with and without DHT, and the P767A mutant with DHT) were extended for 1 microsecond and averaged Cα deviations were calculated from 100 configurations selected at 1 ns intervals from the last 100 ns of these extended trajectories. Several final snapshots were created using the program VMD-1.9.1^21^.

### Statistical analysis

Statistical analysis was performed using GraphPad Prism (GraphPad Software, San Diego CA). All experiment was repeated at least 2 times to confirm reproducibility. Data are provided as the means ± S.D. The significance of difference between control and treated groups was assessed using ANOVA followed by Dunnett’s test for data from multiple groups. The Tukey–Kramer test or Bonferroni’s correction was used to compare multiple groups.

## Results

### AR regulation by Pro767

**Figure 1:**
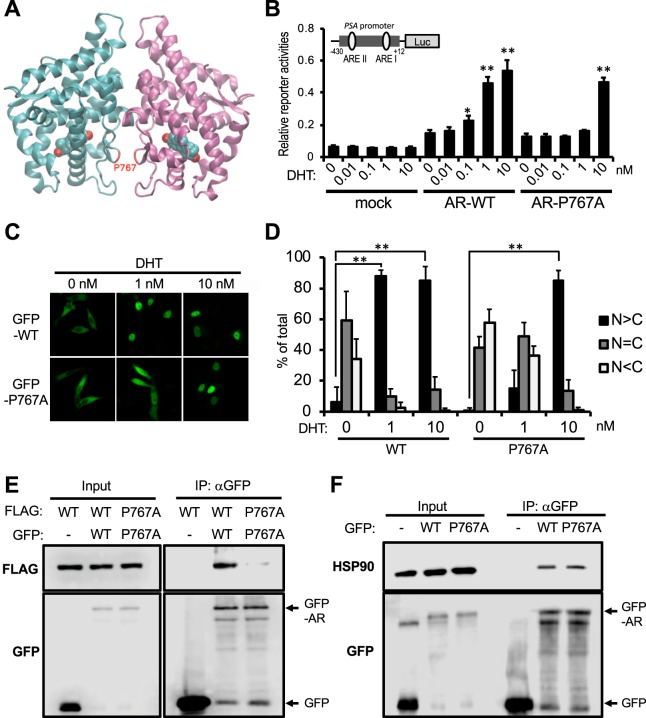
Roles of P767A mutation in AR activation. (**A**) A crystal structure of AR homodimer (pdb id = 5JJM^4^. The sidechain of Pro767 is labeled in red. (**B**) Trans-activation activity. PC-3 cells transfected with PSA-430-pGL3, phRL-TK and expression plasmids for wild type AR (AR-WT) or AR-Pro767Ala were treated with 0.1% DMSO (0 nM) or DHT (0.01, 0.1, 1 or 10 nM) for 24 h. Firefly luciferase activities were normalized with renilla luciferase activities. Values are the mean ±SD (n = 4). *P < 0.05, **P < 0.01 (Dunnett’s test vs 0 nM DHT treated AR-WT expressed group). (**C**) Intracellular localization. PC-3 cells were transfected with expression plasmids for GFP-tagged AR-WT or AR-Pro767Ala mutant and treated with DMSO (0 nM) or DHT (1 or 10 nM) for 1 h. Intracellular distribution was analyzed by a Zeiss LSM 710 with laser excitation lines of 482 nm. (**D**) From each of the three different sets of dishes, over 100 cells in (**C**) were examined to calculate the statistics of AR localizations, predominantly in the nucleus (black bars), equally in the cytoplasm and the nucleus (gray bars), and predominantly in the cytoplasm (white bars). Values are the mean ± SD. **P < 0.01 (Bonferroni’s correction) (**E**) Co-immunoprecipitation. COS-1 cells were co-transfected with FLAG- and GFP-tagged AR WTs or AR Pro767Ala mutants and incubated for 24 h in serum free media, from which whole extracts were prepared and immunoprecipitated with an anti-GFP antibody for subsequent Western blot analysis by an anti-FLAG antibody. (**F**) Co-immunoprecipitation with HSP90. GFP-AR-WT or -Pro767Ala overexpressed in COS-1 cells. Cell extracts were immunoprecipitated by an anti-GFP antibody for subsequent Western blot analysis by an anti-HSP90 antibody.

Nadal *et al*., reposted a crystal structure of DHT-bound AR LBD homodimer structure and performed FRET assays to show ectopically expressed AR LBD formed this homodimer in the nucleus of Hep3B cells^4^. Pro767 resides in the AR homodimer interface and these residues of two monomers interact (Fig. 1A). Pro767Ala mutation disabled AR to form homodimer as observed with wild type AR LBD in their FRET assay^4^. Here we used full-length AR to examine AR homodimerization in the cytoplasm of cells. First, prostate cancer cell line PC-3 cells were expressed with AR WT or AR P767A mutant and treated with 0.1% DMSO as vehicle or 0.01–10 nM DHT for luciferase reporter assay using the promoter of *PSA* gene which includes two AR binding motif^22^. We confirmed that AR P767A mutant required a 10-fold higher DHT concentration to activate PSA promoter-luciferase reporter gene compared to AR WT (Fig. 1B). These different responses correlated with their respective nuclear translocation capabilities; 88% of GFP tagged-AR WT was accumulated in nucleus by 1 nM DHT, while AR P767A translocated into the nucleus by 10 nM but not 1 nM DHT (Fig. 1C,D). FLAG- or GFP-tagged AR WTs and AR P767As were constructed and co-expressed in COS-1 cells, from which extracts were prepared for subsequent co-immunoprecipitation assays with anti-GFP agarose. GFP-AR WT but not GFP precipitated FLAG-AR WT, suggesting AR WT forms homodimer (Fig. 1D). FLAG-and GFP-tagged AR P767A mutants showed a profound decrease of co-precipitation compared with the AR WT (Fig. 1D). Moreover, analysis by 2-dimensional Blue-native/SDS electrophoresis found that AR WT was expressed at a size consistent with AR dimer because it appeared as twice of the monomer size, while the AR P767A mutant was appeared as the monomer (Supplemental Fig. 1). These results indicate that AR WT is retained as a homodimer in the cytoplasm whereas AR P767A mutant is retained as a monomer. One nM DHT induced translocation and transactivation of AR WT homodimer but not AR P767A mutant. AR is known to interact with HSP90 in the cytoplasm^23,24^. Co-immunoprecipitation assays showed, however, that the P767A mutation did not affect AR-HSP90 interactions (Fig. 1F).

### Hormone-induced monomerization and translocation

**Figure 2:**
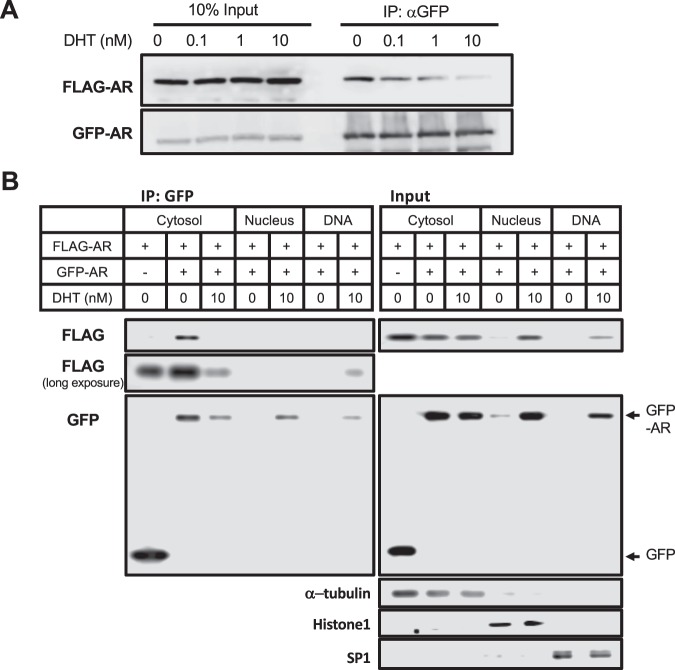
Role of DHT in AR activation. (**A**) Dissociation of AR homodimer. FLAG-AR WT and GFP-AR WT were co-expressed in COS-1 cells and 0.1% DMSO (0 nM) or DHT (0.1, 1 or 10 nM) was treated for 1 hour in serum free media. Whole cell lysate was subjected to co-immunoprecipitation assay with an anti-GFP antibody for subsequent Western blot analysis by an anti-FLAG antibody. (**B**) Dissociation and nuclear translocation. COS-1 were transfected with FLAG- and GFP-tagged AR and treated with 10 nM DHT for 1 hour in serum free media, from which cytosolic, nuclear and DNA-binding fractions were prepared as described in the Methods for subsequent immunoprecipitation by an anti-GFP antibody and Western blot by an anti-FLAG and anti-GFP antibody. Long exposure, Immunoblot by an FLAG-antibody was overexposed.

Given the retention of the AR WT homodimer in the cytoplasm, it was examined whether this dimer dissociated and nuclear translocated in response to DHT. FLAG-tagged AR WT was co-expressed with GFP-tagged AR WT in COS-1 cells and these cells were treated with 0, 0.1, 1 or 10 nM of DHT. As observed in Fig. 1D, FLAG- and GFP-tagged ARs were co-precipitated in the absence of DHT. DHT treatment decreased this co-precipitation in a concentration-dependent manner (Fig. 2A). Thus, AR WT homodimer appeared to dissociate in response to DHT binding. In addition, R1881, a known AR ligand, activated PSA promoter-luciferase reporter gene and, also, dissociated AR homodimer in COS-1 cells (Supplemental Fig. 2A,B). To examine intra-cellular localizations of AR homodimer, FLAG- and GFP-tagged ARs were expressed in COS-1 cells. These cells were fractionated into cytosol, nuclear and DNA binding fractions for subsequent co-immunoprecipitation and Western blot assays (Fig. 2B). As inputs suggested, AR was expressed in cytosolic fractions and accumulated in nuclear and DNA-binding fractions. Cytosolic AR levels decreased after 10 nM DHT treatment, reciprocally, nuclear AR levels increased. Co-immunoprecipitation assays revealed that cytosolic GFP- and FLAG-ARs co-precipitated in the absence of DHT (Fig. 2B). AR accumulated in the nucleus only when cells were treated with 10 nM DHT. GFP- and FLAG-ARs in nuclear fractions did not co-precipitate (Fig. 2B). On the other hand, ARs in DNA binding fractions were not co-precipitated (Supplemental Fig. 6). Long-exposure of membrane showed FLAG-AR precipitation in the DNA binding fractions. These observations suggested that ectopic AR formed a homodimer in the cytoplasm in absence of DHT and accumulated as its monomer in the nucleus after DHT treatment, then reforming homodimer to bind DNA.

**Figure 3:**
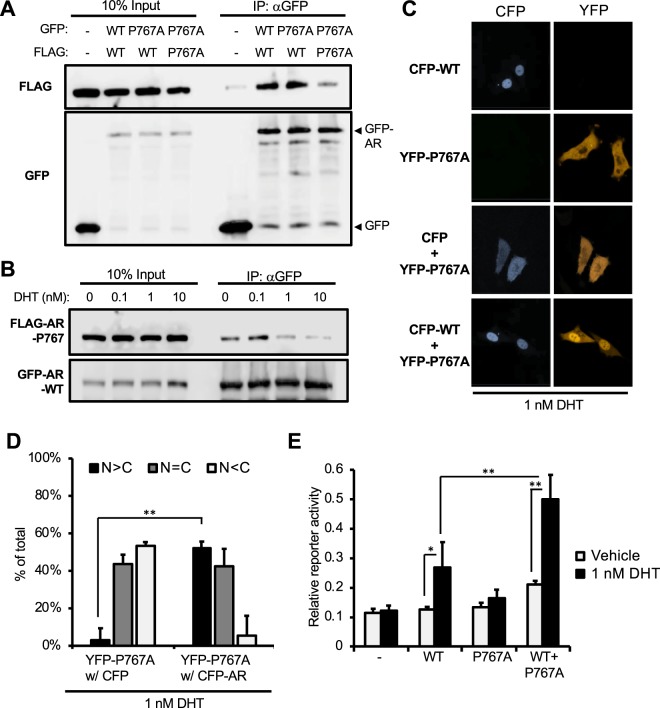
AR-Pro767Ala heterodimer with AR WT and nuclear translocation. (**A**) Immunoprecipitation of heterodimer. COS-1 cells co-transfected with FLAG- or GFP-AR Pro767Ala and -AR WT in combinations as indicated and incubated for 24 h in serum free media, from which whole extracts were prepared for subsequent immunoprecipitation and Western blot with an anti-GFP antibody and an anti-FLAG antibody. (**B**) Heterodimer dissociation; COS-1 cells were co-expressed with FLAG-AR Pro767Ala and GFP-AR WT and treated by DHT (0, 0.1, 1 or 10 nM) for 1 hour, from which whole extracts were prepared for subsequent immunoprecipitation and Western blot analysis. (**C**) Intracellular localization: PC-3 cells expressed CFP-AR-WT (CFP-WT) or YFP-AR Pro767Ala (YFP-P767A) or co-expressed CFP and YFP-AR Pro767Ala or CFP-AR WT and YFP-AR Pro767Ala were treated with 1 nM DHT for 1 h. Cellular distributions were analyzed a Zeiss LSM 710. (**D**) Localization of YFP-AR Pro767Ala were counted. Over 100 cells of each dishes (three dishes per each samples) in (**C**) were examined to calculate the statistics of AR localization, predominantly in the nucleus (black bars), equally in the cytoplasm and the nucleus (gray bars), and predominantly in the cytoplasm (white bars). Values are the mean ± SD. **P < 0.01 (Bonferroni’s correction) (**E**) Trans-activation activity: PC-3 cells were transfected with PSA-pGL3, phRL-TK and expression plasmid for AR-WT or AR-Pro767Ala or with both and treated with 0.1% DMSO or 1 nM DHT for 24 h. Firefly luciferase activities were normalized with renilla luciferase activities. Values are the mean ± SD (n = 4). *P < 0.05, **P < 0.01 (Tukey–Kramer test).

AR Pro767Ala didn’t form a homodimer and retained in the cytoplasm at 1 nM DHT (Fig. 1). GFP-tagged AR Pro767Ala was co-expressed with FLAG-tagged AR WT in COS-1 cells to examine the capabilities of this AR mutant to form a heterodimer and to nuclear translocate. GFP-AR Pro767Ala was co-immunoprecipitated with FLAG-AR WT, suggesting that they formed a heterodimer (Fig. 3A). This heterodimer dissociated in a DHT like that observed with the AR WT homodimer (Fig. 3B). Confocal analysis showed that YFP-AR Pro767Ala significantly translocated in the nucleus by 1 nM DHT when co-expressed with CFP-AR WT (Fig. 3C,D). In this co-expression, AR Pro767Ala stimulated AR-WT dependent PSA promoter activities (Fig. 3E).

**Figure 4:**
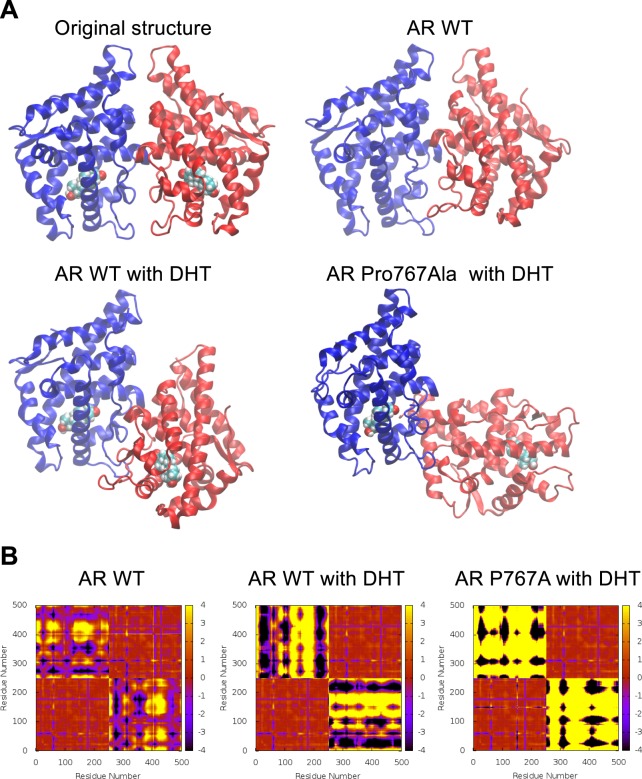
Dynamic simulation of AR homomer. (**A**) Snapshots of simulated structures at 1 microsecond. Comparison of monomer arrangements in various dimer systems; one monomer (monomer A) is displayed in blue and the other (monomer B) in red. The blue monomer of each system is aligned to capture the relative displacements. (**B**) Cα deviations averaged over the last 100 ns of molecular dynamics. Red displays no or smaller deviations from the starting distances and black and yellow correspond to the maximum deviations to one direction or the other. Residue numbers 1 to 251 belong to monomer A and 252 to 502 belong to monomer B. Bottom left and top right quadrants represent intra-monomer deviations of monomers A and B, respectively. Top left or bottom right quadrants represent inter-monomer deviations between the monomers A and B. Each figure is symmetric around the diagonal passing through (0,0).

Molecular dynamic (MD) simulations were performed on AR homodimer with and without DHT binding (Fig. 4). In the X-ray crystal structure of DHT-bound AR homodimer, two monomers interact symmetrically. To reveal monomer arrangements from MD, the homodimer structures were captured at 1 microsecond and were compared with the X-ray structure (Fig. 4A). In the WT AR without DHT, the monomers appeared to retain symmetry relative to one another. On the other hand, the AR monomers bound to DHT increased asymmetry, indicating that DHT binding destabilized the homodimer. As expected, the DHT-bound AR P767Ala mutant completely lost the symmetry. This mobility was well established from the movies provided in Supplemental Information (Supplemental Movie 1). As these AR homodimers increased asymmetry, Cα deviations representing inter-monomer distances became larger as clearly visible from the plots in Fig. 4B (regions representing inter-domain deviations displayed increased black or yellow areas and decreased red areas).

### Regulation by Phe765, Met743 and Trp742

**Figure 5:**
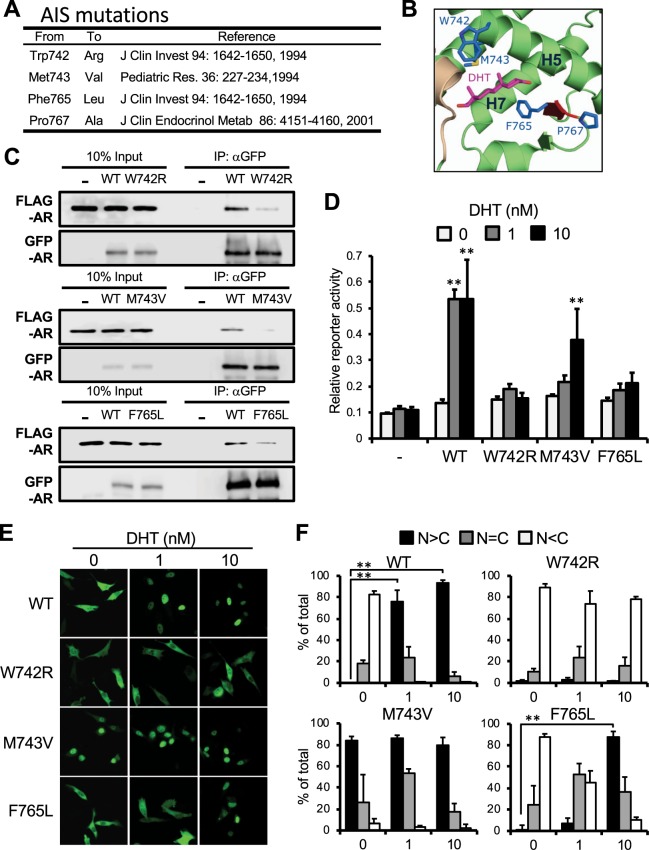
Roles of Phe765 and Met743 in AR activation. (**A**) Selected AIS-associated mutations are described. (**B**) Sidechains of these residues are mapped in the DHT-bound AR LBD homodimer structure. (**C**) Homodimerization: COS-1 cells co-expressed with FLAG-AR WT and GFP-AR WT or with a given FLAG- and GFP-AR mutants, from which whole extracts were prepared for subsequent immunoprecipitation and Western blot analysis as described in Fig. 1E. (**D**) Trans-activation activity; PC-3 cells were transfected with PSA-pGL3, phRL-TK and an expression plasmid for AR-WT, -Trp742Arg, -Met743Val or -Phe765Leu and were treated with 0.1% DMSO or 1 or 10 nM DHT for 24 h. Firefly luciferase activities were normalized with renilla luciferase activities. Values are the mean ±SD (n = 4). **P < 0.01 (Dunnett’s test vs 0 nM DHT treated AR-WT expressed group). (**E**) Intracellular localization; PC-3 cells expressed with the GFP-AR-WT, -Trp742Arg, -Met743Val or -Phe765Leu, treated with 0.1% DMSO (0 nM) or 1 or 10 nM DHT for 1 h. and green fluorescence was analyzed by a Zeiss LSM 710 with laser excitation lines of 482 nm. (**F**) From each of the three different sets of dishes, over 100 cells in Fig. 5E were examined to calculate the statistics of AR localizations, predominantly in the nucleus (black bars), equally in the cytoplasm and the nucleus (gray bars), and predominantly in the cytoplasm (white bars). Values are the mean ±SD. **P < 0.01 (Bonferroni’s correction).

Mutations Trp742Arg, Met743Val and Phe765Leu have been reported to be associated with AIS (Fig. 5A). These Trp, Met and Phe residues are located on the surface of the ligand binding pocket of AR LBD structure. Phe765 resides on a short β-strand near the loop containing Pro767 and sandwiches with Met743 (Fig. 5B). Subsequently, these mutations were examined as to whether they altered homodimerization and nuclear translocation of AR in cells. Whole cell extracts were prepared for co-immunoprecipitation assays. Like the AR Pro767Ala mutant, these three mutants were poorly co-precipitated compared with the AR WT (Fig. 5C). Confocal analysis of GFP-tagged AR revealed that the AR Phe765Leu mutant was retained in the cytoplasm at 1 nM DHT and translocated in the nucleus at 10 nM DHT, closely resembling the Pro767A mutant (Fig. 5E,F). AR Trp724Arg did not translocate even at 10 nM DHT, while AR Met743Val spontaneously translocated into the nucleus in the absence of DHT(Fig. 5E,F). In reporter assays, AR Phe765L did not activate a PSA promoter even at 10 nM DHT although it was in the nucleus at this concentration (Fig. 5D). AR Met743Val activated the promoter less effectively than the AR Pro767Ala mutant. Consistent with its nuclear localization, the AR Trp742Arg mutant was unable to activate the promoter. These three AR mutants exhibit similar DHT binding free energies similar to those of AR WT and AR Pro767Ala mutant, with the exception of Trp42Arg which has a slightly more positive binding free energy (Table 1).

**Table 1:** DHT binding energy of MD simulations.

Mutation	DHT binding energy (kcal/mol)
WT	−22.6 ± 3.1
W742R	−15.4 ± 3.3
M743V	−20.5 ± 3.3
F765L	−21.7 ± 3.5
P767A	−21.4 ± 3.3

### GR regulation by Phe623 and Met601

**Figure 6:**
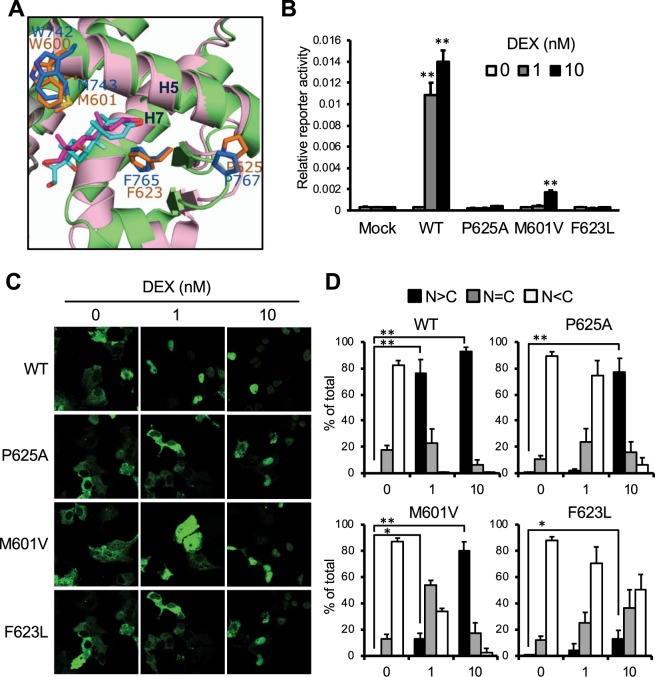
Roles of the corresponding residues in GR activation. (**A**) Superimposing between crystal structures of GR LBD and AR LBD (green); Pro765, Phe765, Met632 of AR are superimposed with Pro625, Phe623 and Met601 of GR, respectively. (**B**) Trans-activation activity; COS-1 cells co-transfected with GRE-pGL3, phRL-TK and expression plasmid for GR-WT, -Pro625Ala, -Met601Val or -Phe623Leu were treated with 0.1% DMSO (0 nM) or DEX (1 or 10 nM) for 24 h. Firefly luciferase activities were normalized with renilla luciferase activities. Values are the mean ±SD (n = 4). **P < 0.01 (Dunnett’s test vs 0 nM DHT treated AR-WT expressed group). (**C**) Intracellular localization; COS-1 cells transfected with an expression plasmid for CFP-GR-WT, -Pro625Ala, -Met601Val or -Phe623Leu were treated with 0.1% DMSO or 1 or 10 nM DEX as and analyzed above-described. (**D**) From each of the three different sets of dishes, over 100 cells in (**C**) were examined to calculate the statistics of AR localizations, predominantly in the nucleus (black bars), equally in the cytoplasm and the nucleus (gray bars), and predominantly in the cytoplasm (white bars). Values are the mean ±SD. **P < 0.01, *P < 0.05 (Bonferroni’s correction).

Pro767 of AR is superimposed with Pro625(conserved residues) in the GR (Fig. 6A). As Pro767 at the AR interface, Pro625 residues of GR monomers interact in the homodimer interface^11^. The Pro625Ala mutation was previously examined as a regulator of GR homodimerization in the 1990’s. Recombinant GR and its mutant proteins were analyzed *in vitro* by density gradient ultracentrifugation^11,25^. In addition to Pro767, Phe765 and Met743 of AR are superimposed with the conserved residues, Phe623 and Met601, respectively, in GR (Fig. 6A). To examine roles of these residues in GR activity, Pro624, Phe623 and Met601 of GR were mutated to Ala, Leu and Val, respectively; GR Pro625Ala, GR Phe623Leu and GR Met601Val. Cell-based reporter assays with GRE-luciferase gene showed these mutants were unable to activate the reporter gene even at 10 nM DEX while GR WT fully activated it at 1 nM DEX (Fig. 6B). Moreover, these GFP-tagged GR mutants expressed in Huh-7 were retained in the cytoplasm even after treatment with 10 nM DEX (Fig. 6C,D).

## Discussion

Ligand-bound homodimerization in the nucleus has been a major focus of AR research (1). On the other hand, AR in the cytoplasm was not well-studied. A recent structure of AR LBD homodimer revealed that AIS-associated mutations are found on a surface of the homodimer interface (D3L), although many of them are located within the ligand-binding pocket (LBP)^26^. Among these former mutations, Pro767Ala and Phe765Leu are intriguing since both co-localize in a loop that constitutes the homodimer interface. Pro767 directs its sidechain towards a surface of the other monomer, while the sidechain of Phe765 interacts with the DHT molecule in the LBP. Phe765 may be a molecular switch endowing DHT binding to Pro767, the regulator of AR homodimerization. Our present works showed that AR is expressed as a homodimer in the cytoplasm and that DHT binding induces translocation of the AR monomer into the nucleus. The homodimerization and nuclear translocation events appear to link the DHT response in AR activation.

The reported AR LBD homodimer crystal structure contains the DHT molecule (4). In FRET assays, ectopically- expressed AR LBD molecules interacted through Pro767 in the nucleus (4). Consistent with a general knowledge, this AR homodimer was considered as a DHT-liganded homodimer that binds DNA. However, the FRET assays of AR lacking the DBD could not detect a DNA-bound form of AR homodimer. On the other hand, our present study utilized a full-length AR molecule that is properly retained in the cytoplasm, translocated to the nucleus and activated genes in response to DHT in cells. This reported AR LBD homodimer is partially restrained through the D3L interface by Van der Waals interactions between the Pro767 residues of the two AR monomers. In fact, weakening of this interaction by replacing Pro with Ala disables the AR to homodimerization. On the other hand, the AR Pro767Ala mutant was co-immunoprecipitated with AR WT co-expressed in COS-1 cells. Thus, a subtle change of free binding energy associated with a conformational alteration of Pro767 may be sufficient to support dimerization. MD simulation was performed to display molecular motions of AR WT homodimer with or without DHT and of AR Pro767Ala mutant with or without DHT (Supplemental Movie 1). DHT binding appeared to increase motions which separate the AR homodimer in both the WT and mutant. Therefore, it is reasonably expected that, upon Ala mutation and/or DHT binding, Phe765 causes a subtle conformational change of Pro767, thereby dissociating the AR homodimer. While the Phe765 sidechain directly interacts with the DHT molecule which places it in the potion to transduce DHT binding signal to Pro767, Phe765 does not interact with Pro767. Instead, the side chain of the Tyr764 is directed towards Pro767, thereby forming a Van der Waals interaction (Supplemental Fig. 3). Thus, Tyr764, Phe765 and Pro767 form a triangular configuration of interactions (Supplemental Fig. 3). Phe765 may support DHT binding to Pro767 through Tyr764, dissociation of the AR homodimer in the cytoplasm. Met743 directly interacts with the DHT molecule, sandwiching it with Phe765. Thus, the Pro-Phe-Met may form the relay to induce the DHT binding signal. Tyr764 of AR is conserved in PR and MR, but not in GR.

Unlike the AR LBD that has been suggested to form a homodimer in response to DHT in the nucleus^4^, the full-length AR did not form a homodimer before DNA binding in the nucleus. A scenario that AR undergoes in response to DHT is depicted. DHT binding dissociates the AR homodimer in cytoplasm. Resulted DHT-bound AR monomer may form N/C terminal interactions^27^ to translocate in the nucleus. Such N/C interactions are also known to regulate AR dimerization, DNA binding or transcription^28–31^. AR’s Pro767 residue is known to be critically involved in regulating a DBD-LBD interaction of the AR monomer^32^. Although these intra- as well as intermolecular interactions have primarily been related to AR in the nucleus, our findings indicate that AR also undergoes these interactions in cytoplasm. In addition to AR, GR homodimer utilizes its D3L interface^11^, and progesterone receptor (PR), which is reported to form homodimer in solution without DNA binding motif^33^, utilizes different interface from ER or HNF4α^34–36^. Thus ligand-induced monomerization maybe a regulatory process common to steroid hormone receptors. Our co-immunoprecipitation experiments also suggest that, once DHT-liganded AR binds DNA, it does so as a homodimer. Whether this AR homodimer utilizes the D3L interface remains as an important subject for future investigations. However, it is not unreasonable to think that the AR homodimer which dissociates by DHT in the cytoplasm is stabilized by DNA binding with transcription coregulators, in the nucleus.

Homodimerization/monomer conversion appears linked to proper nuclear translocation of AR. Among ARs examined, only AR WT underwent dissociation and translocation. All four mutants (AR Pro767Ala, Phe765Leu, Met743Val and Trp742Arg) neither formed a homodimer nor properly translocated; AR Met743Val spontaneously translocated while the other three mutants remained in the cytoplasm at 1 nM DHT. In support of this proposed link, AR Pro767Ala co-expressed with AR WT formed a heterodimer and dissociated at 1 nM DHT, translocating into the nucleus. Thus, heterodimerization with AR WT enabled AR Pro767Ala to translocate in the nucleus in response to ligand and induce transcription of its target reporter gene. Similar to AR, GR was reported that it mutant restored nuclear translocation capability by heterodimerizing with wild type MR^37^. We demonstrated that nuclear translocation of AR depends on its ligand-induced homodimerization in the cytoplasm. Similarly, cytoplasmic GR homodimer was reported to dissociate in response to by a plant delivered ligand compound A^38^.

Thus, ligand-free homodimerization appears to be prerequisite for AR translocation. Heterodimerization with AR wild-type restored AR P767A’s lost-functions. Thus, heterozygous mutants might not be defective in AIS patient. However, AR is encoded by a single copy X-chromosomal gene, suggesting heterozygous AR mutants shouldn’t exist in male sex characterized AIS patient.

GR also underwent ligand-free homodimerization in the cytoplasm and DEX response monomerization and subsequent nuclear translocation. Consistent with AR and GR, the nuclear receptor CAR formed a homodimer via the D3L interface in the cytoplasm, dissociation of the homodimer and subsequent translocation to the nucleus^12,13^.

GR conserves these residues corresponding to Pro767, Phe765 and Met743 of AR as Pro625, Phe623 and Met601 (Fig. 6A). Pro625 was previously shown to regulate GR homodimerization^11^. Now, our present study has found that the corresponding mutations (Pro625Ala, Phe623Leu and Met601Val) disabled GR from responding to DEX as observed with AR mutants to DHT. Moreover, the Pro-Phe-Met axis is conserved in mineralocorticoid receptor (MR) and PR (Supplemental Fig. 4). It is anticipated that future investigation will establish the axis as a structural motif that induces a ligand-binding signal that regulates homodimerization of steroid hormone receptors. In conclusion, this apparent conservation of the Pro-Phe-Met relay regulating cytoplasmic homodimerization and ligand-induced monomerization, nuclear translocation and promoter activation suggests that steroid hormone receptors have evolved by conserving the same structural motif for hormonal activation. Acting as a molecular switch, the Phe residue may play the most unique role in transmitting the steroid binding signal to the Pro residue for homodimer dissociation. The discovery of the cytoplasmic homodimer may lead us to be able to characterize non-genomic functions of steroid hormone receptors in future investigations.

## Supplementary information

Supplemental Figure1-8

Supplemental movie

## Data Availability

The datasets generated and analyzed during the current study are available from the corresponding author on reasonable request.
