# Supplementary material for: Ligand induced dissociation of the AR homodimer precedes AR monomer translocation to the nucleus
Source: Sci Rep. 2019 Nov 13;9:16734. doi: 10.1038/s41598-019-53139-9 (PMC6853983; doi:10.1038/s41598-019-53139-9)

Supplementary Information

Ligand induced dissociation of the AR homodimer precedes AR monomer translocation to the nucleus

Ryota Shizu, Kosuke Yokobori, Lalith Perera, Lee Pedersen, Masahiko Negishi
Pharmacogenetic section, Reproductive and Developmental Biology Laboratory and Genome Integrity and Structural Biology Laboratory, National Institute of Environmental Health Sciences, National Institutes of Health, Research Triangle Park, North Carolina, 27709, USA

Supplemental figure 1. Blue-native/SDS PAGE two-dimensional electrophoresis.
Cell lysates were prepared from the PC-3 cells overexpressed GFP-tagged-AR WT or Pro767Ala. A GFP antibody was used for Western blot to detect AR. Molecular weights (kDa) for Blue-native PAGE are indicated above gels.

Supplemental figure 2. R1881 dissociates the interaction of ARs.
(A) COS-1 cells transfected with PSA-430-pGL3, phRL-TK and expression plasmids for wild type AR (AR-WT) were treated with 0.1% DMSO (0 nM) or R1881 (0.01, 0.1, 1 or 10 nM) for 24 h. Firefly luciferase activities were normalized with renilla luciferase activities. Values are the mean ±SD (n = 4). *P <0.05, **P <0.01 (Dunnett's test vs DMSO treated AR-WT expressed group). (B) COS-1 cells were co-expressed with FLAG-AR WT and GFP-AR WT and treated with 0.1% DMSO (0 nM) or R1881 (0.01, 0.1 or 1 nM) for 1 hour. Whole cell lysate was subjected to co-immunoprecipitation assay as described in the legend of Fig. 2A.

Supplemental figure 3. Displayed residues Pro767 and Met743 are implied in AIS-associated mutations. Pro767 is in the dimer interacting surface along with Tyr764. The two proline residues from each monomer are in van der Waals contacts with each other (closest atoms are separated only by 2.4A). The residues on the left of the horizontal arrow in the middle belong to one monomer and those on the right belong to the other monomer. DHT molecules are shown in the solid filled representation.

Supplemental figure 4. Superposition of the Pro-Phe-Met axis.
AR (5JJM in green) was superimposed with GR (1M2Z in pink), PR (1A28 in yellow) and MR (2AA5 in blue). Ligands bound are 5-alpha-dihydrotestosterone, dexamethasone, progesterone and progesterone, respectively. Numbers are residues of AR. These corresponding residues are Pro625, Phe623 and Met601 in GR, Pro780, Phe778, Met756 in PR and Pro831, Phe829, Met807 in MR.

Supplemental figure 5. Full-length gel images of Fig. 1.
(A) Full-length gel images of Fig. 1E. (B) Band intensities of Fig. 1E were quantified using ImageJ analysis software. (C) Full-length gel images of Fig. 1F. (D) Band intensities of Fig. 1F were quantified using ImageJ analysis software.

Supplemental figure 6. Full-length gel images of Fig. 2.
(A) Full-length gel images of Fig. 2A. (B) Band intensities of Fig. 2A were quantified using ImageJ analysis software. (C) Full-length gel images of Fig. 2B. (D) Band intensities of Fig. 2B were quantified using ImageJ analysis software.

Supplemental figure 7. Full-length gel images of Fig. 3.
(A) Full-length gel images of Fig. 3A. (B) Band intensities of Fig. 3A were quantified using ImageJ analysis software. (C) Full-length gel images of Fig. 3B. (D) Band intensities of Fig. 3B were quantified using ImageJ analysis software.

Supplemental figure 8. Full-length gel images of Fig. 5.
(A) Full-length gel images of Fig. 5C. (B) Band intensities of Fig. 5C were quantified using ImageJ analysis software.

Supplemental movie 1. Equally spaced configurations at every nanosecond were selected to create the movie showing the dimer dynamics of AR dimer.
(A) with no DHT-bound form, (B) in the DHT-bound form, and (C) in the DHT-bound form with Pro767Ala mutations.

Supplemental Fig. 1

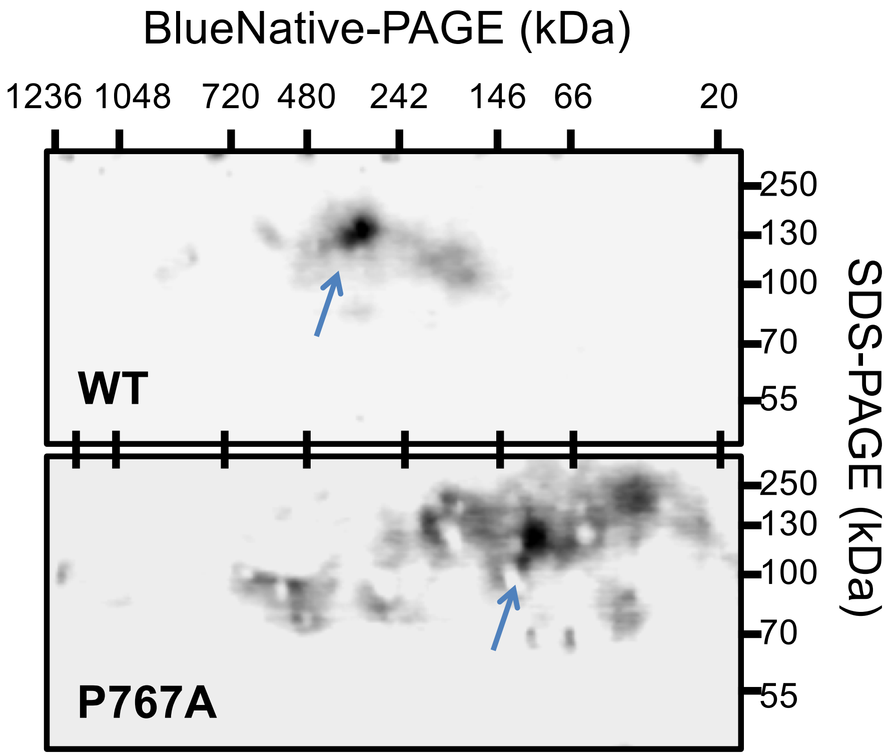

Supplemental Fig. 2

A

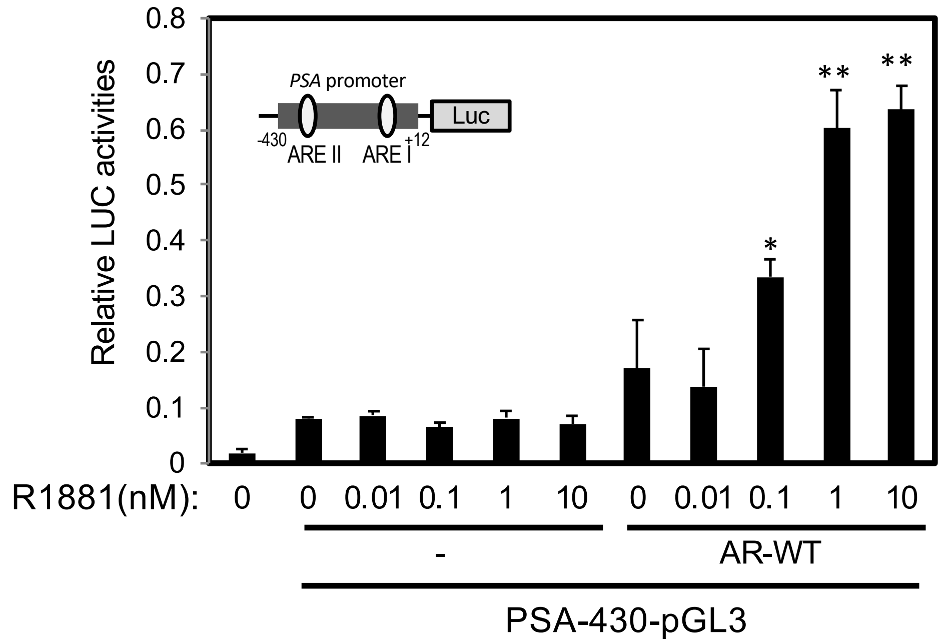

B

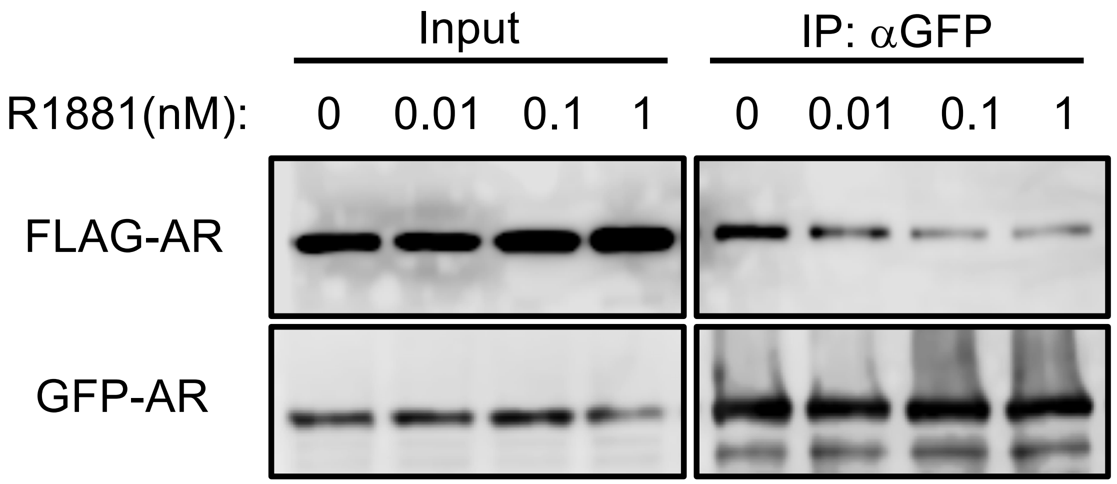

Supplemental Fig. 3

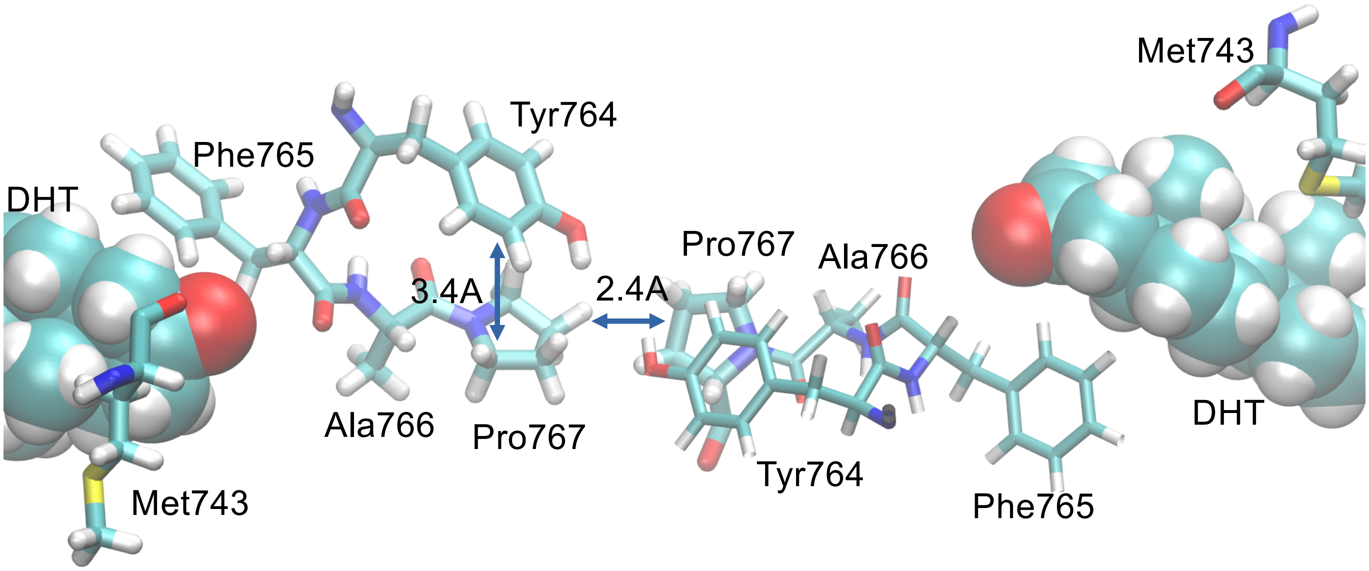

Supplemental Fig. 4

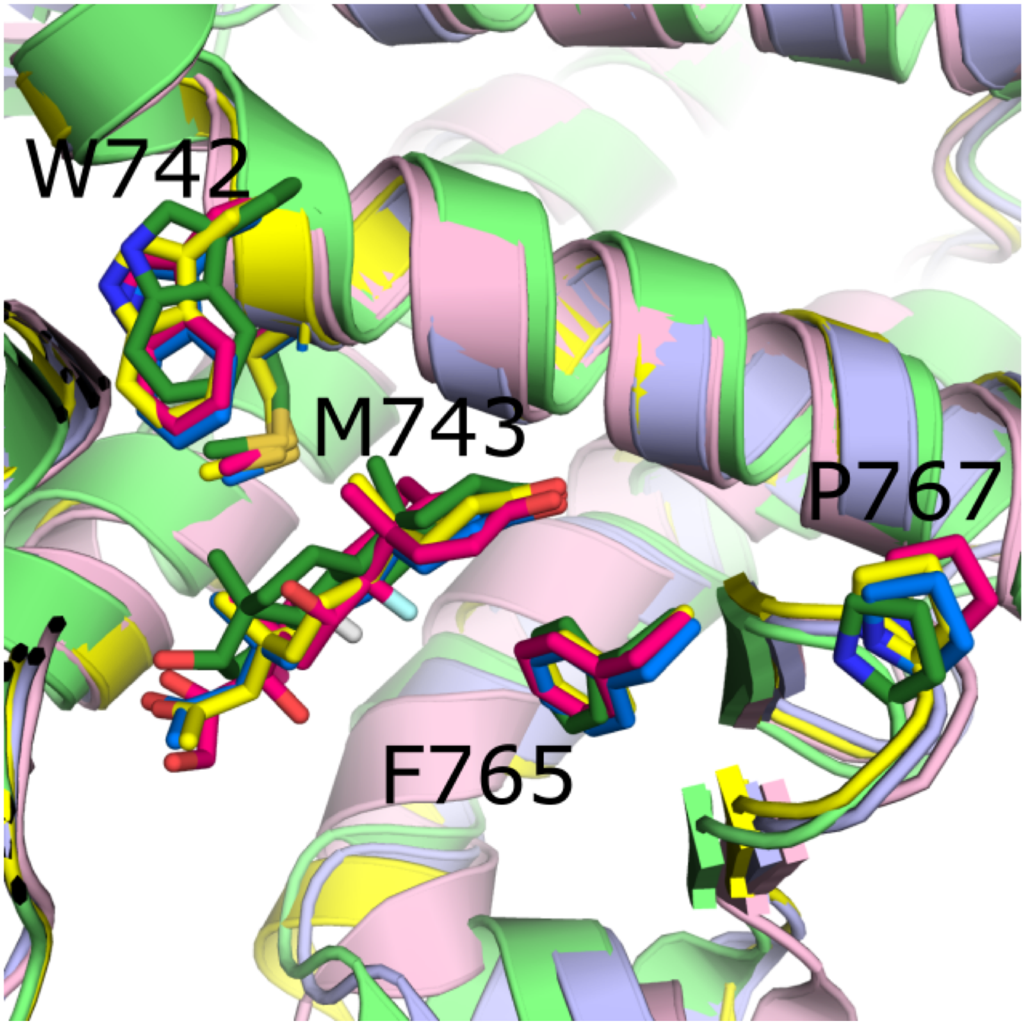

W-M-$(X)_{22}$-F-X-P

Supplemental Fig. 5

A

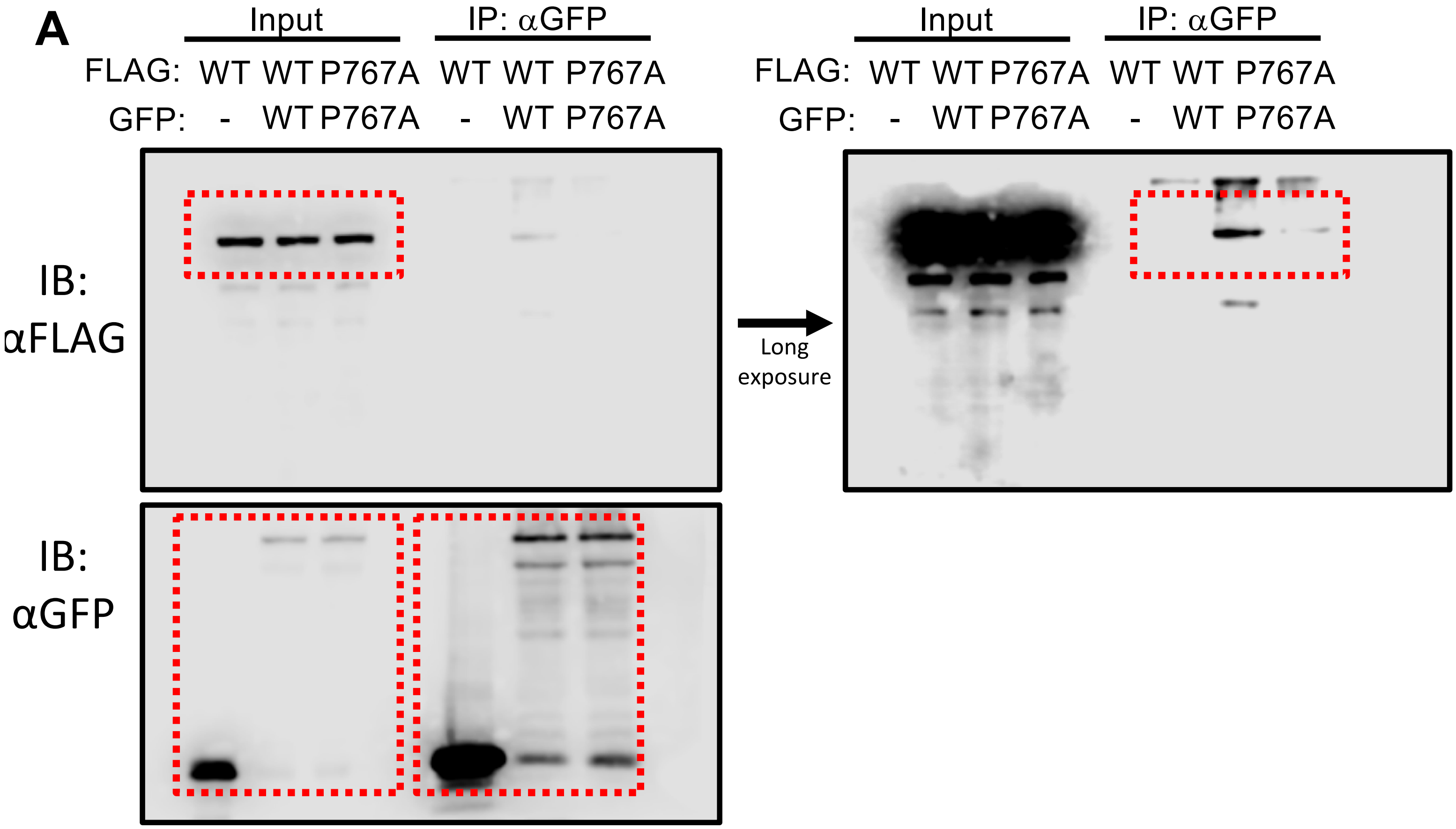

B

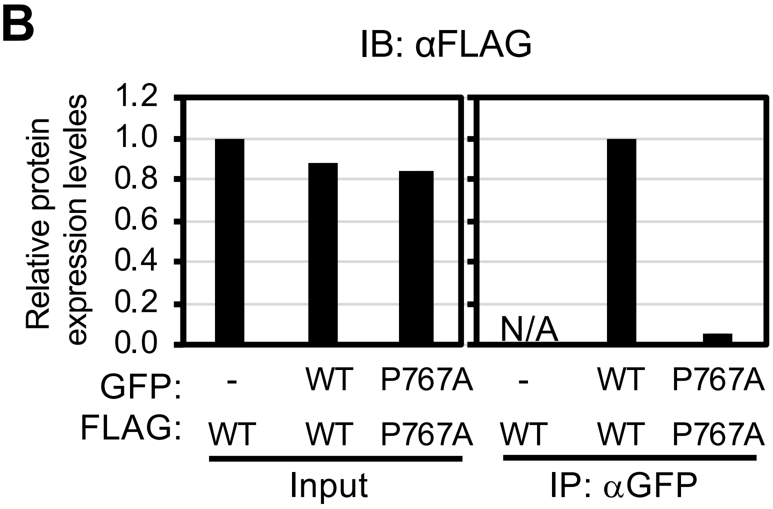

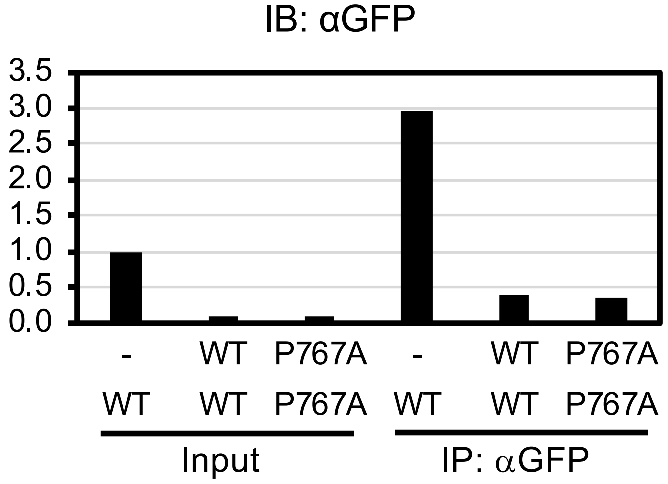

Supplemental Fig. 5

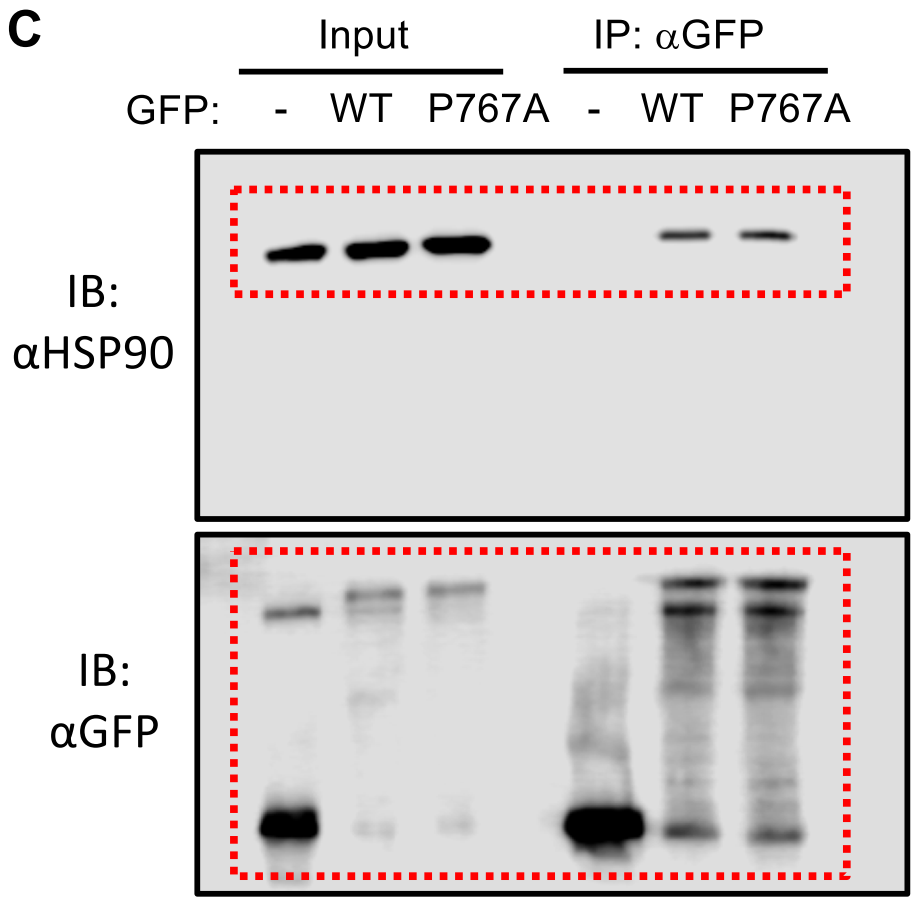

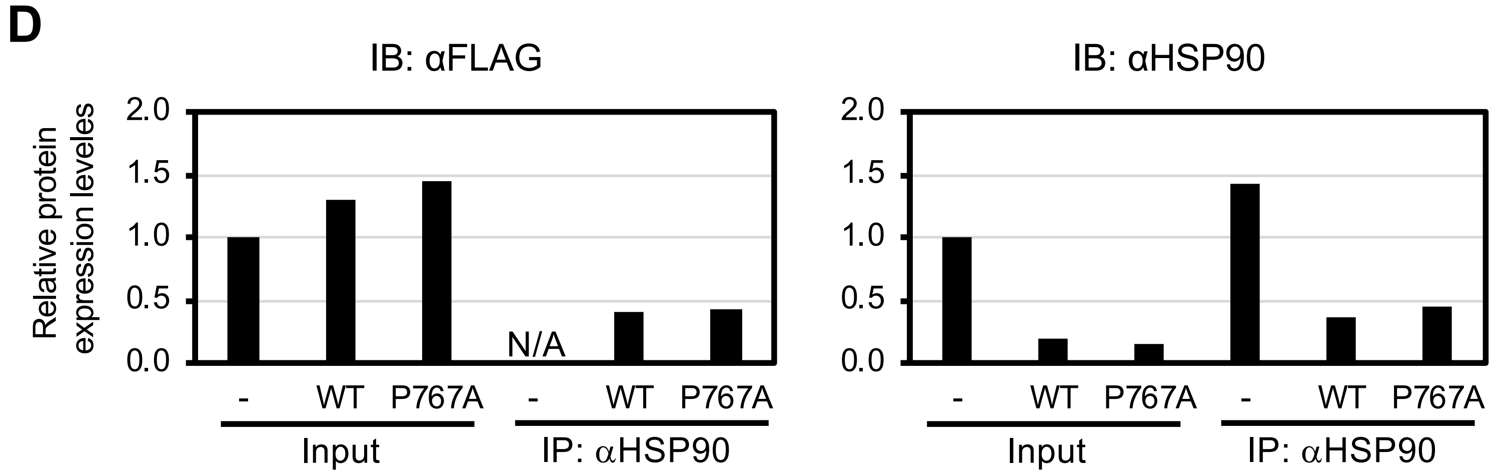

Supplemental Fig. 6

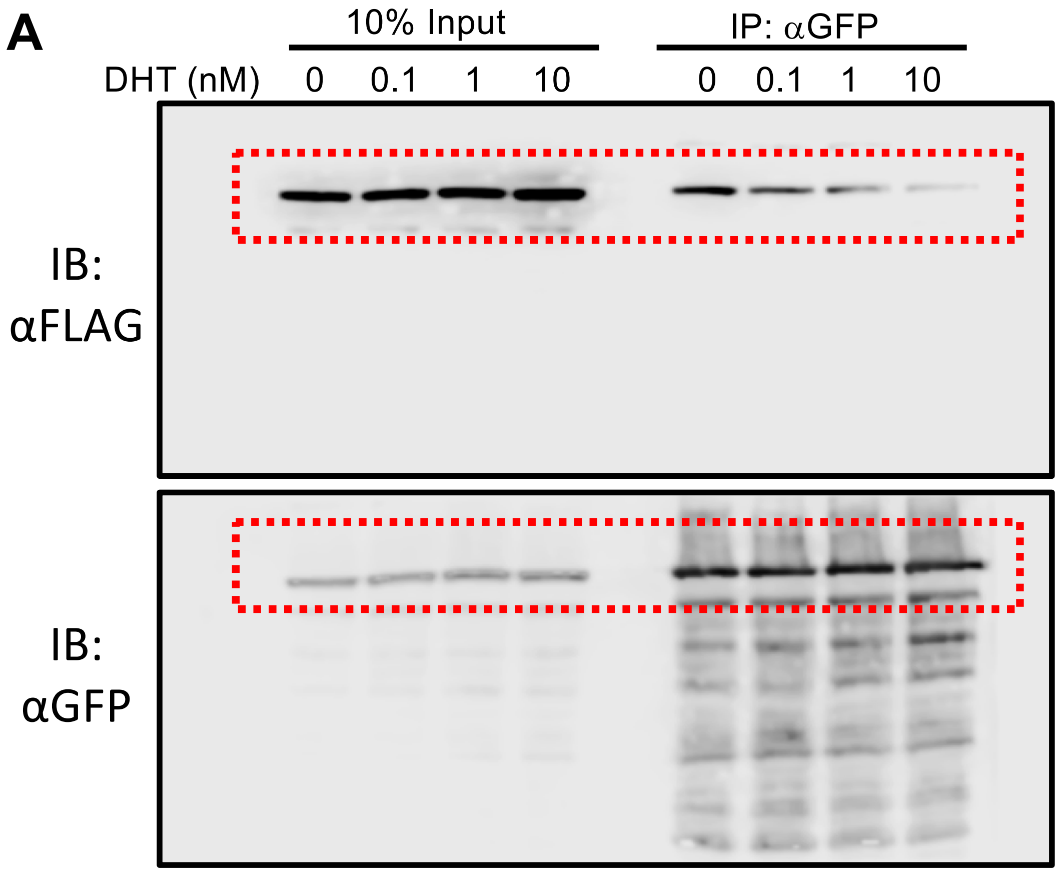

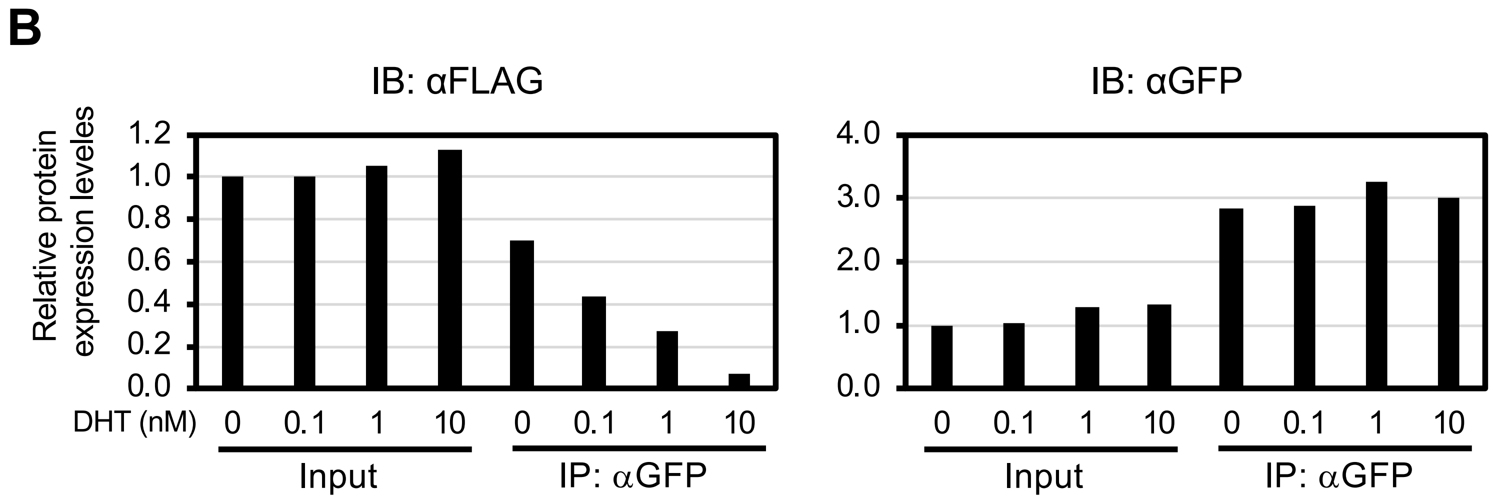

Supplemental Fig. 6

C

IP: GFP

	Cytosol			Nucleus		DNA	
FLAG-AR	+	+	+	+	+	+	+
GFP-AR	-	+	+	+	+	+	+
DHT (nM)	0	0	10	0	10	0	10

Input

	Cytosol			Nucleus		DNA	
FLAG-AR	+	+	+	+	+	+	+
GFP-AR	-	+	+	+	+	+	+
DHT (nM)	0	0	10	0	10	0	10

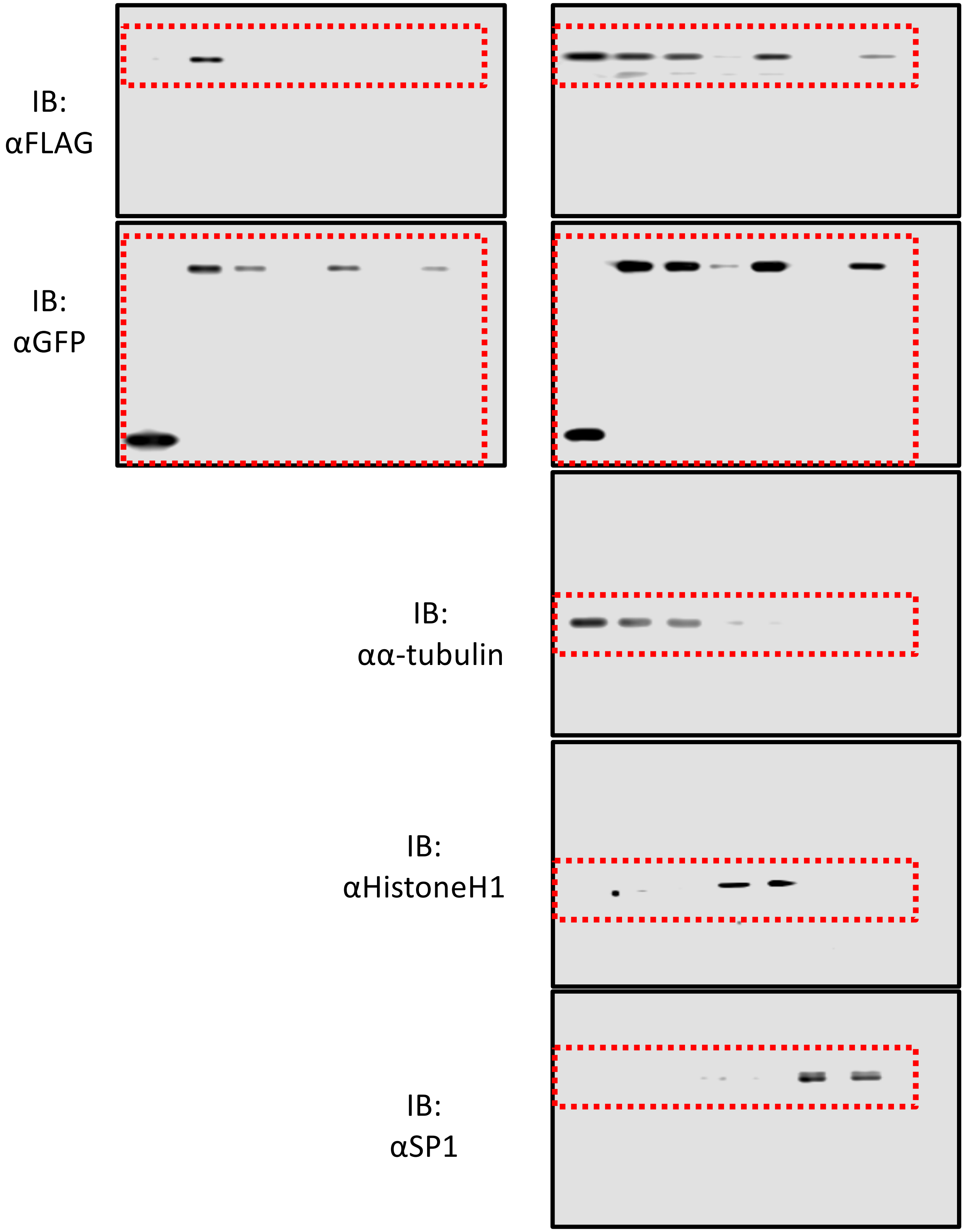

Supplemental Fig. 6

D

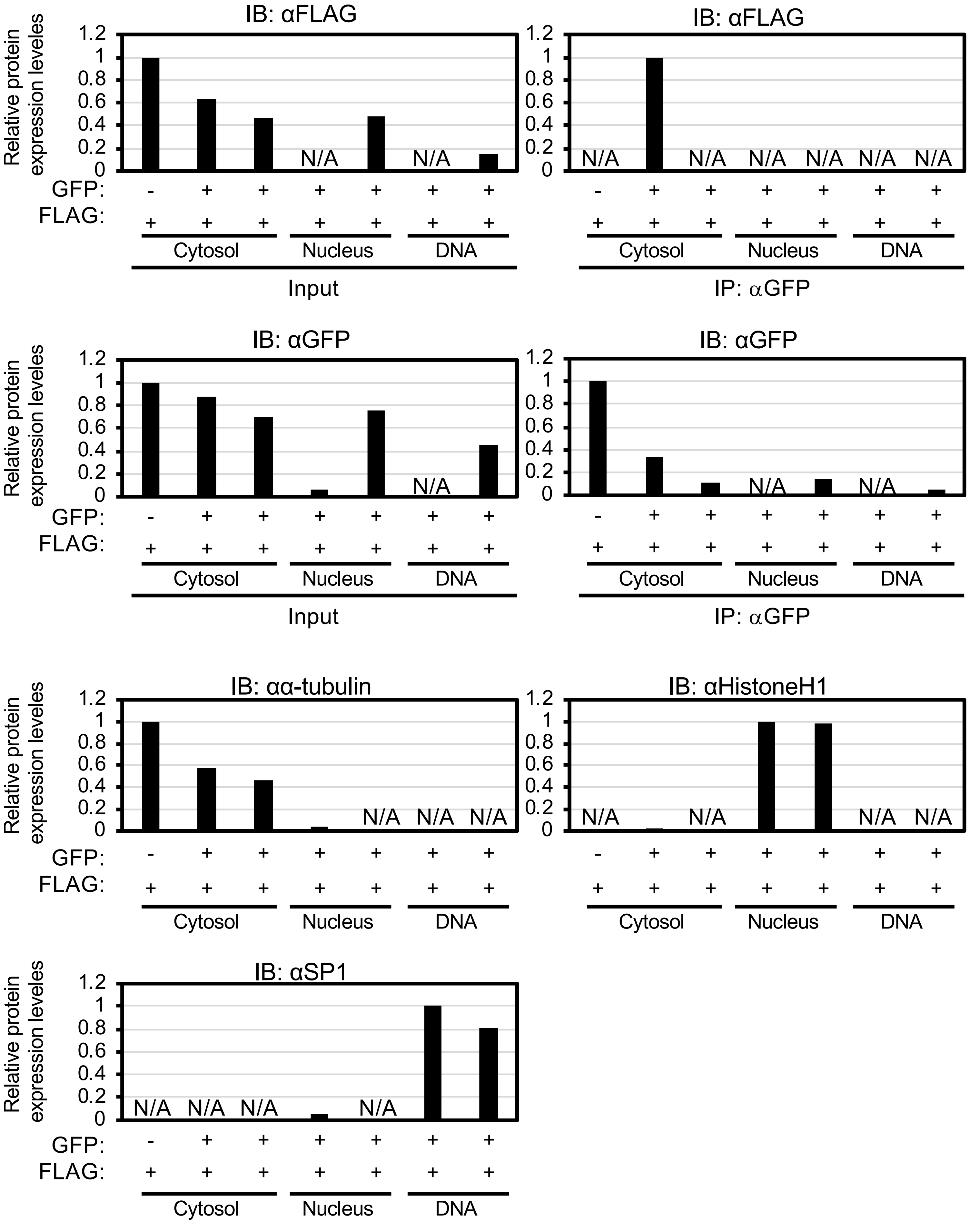

Supplemental Fig. 7

A

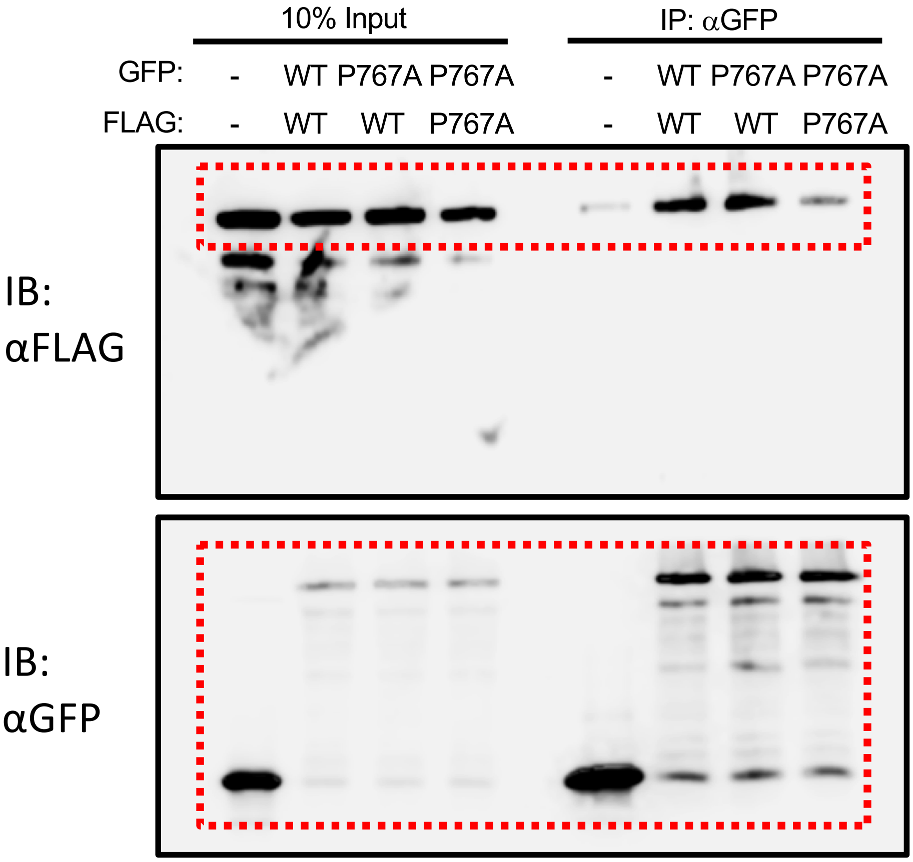

B

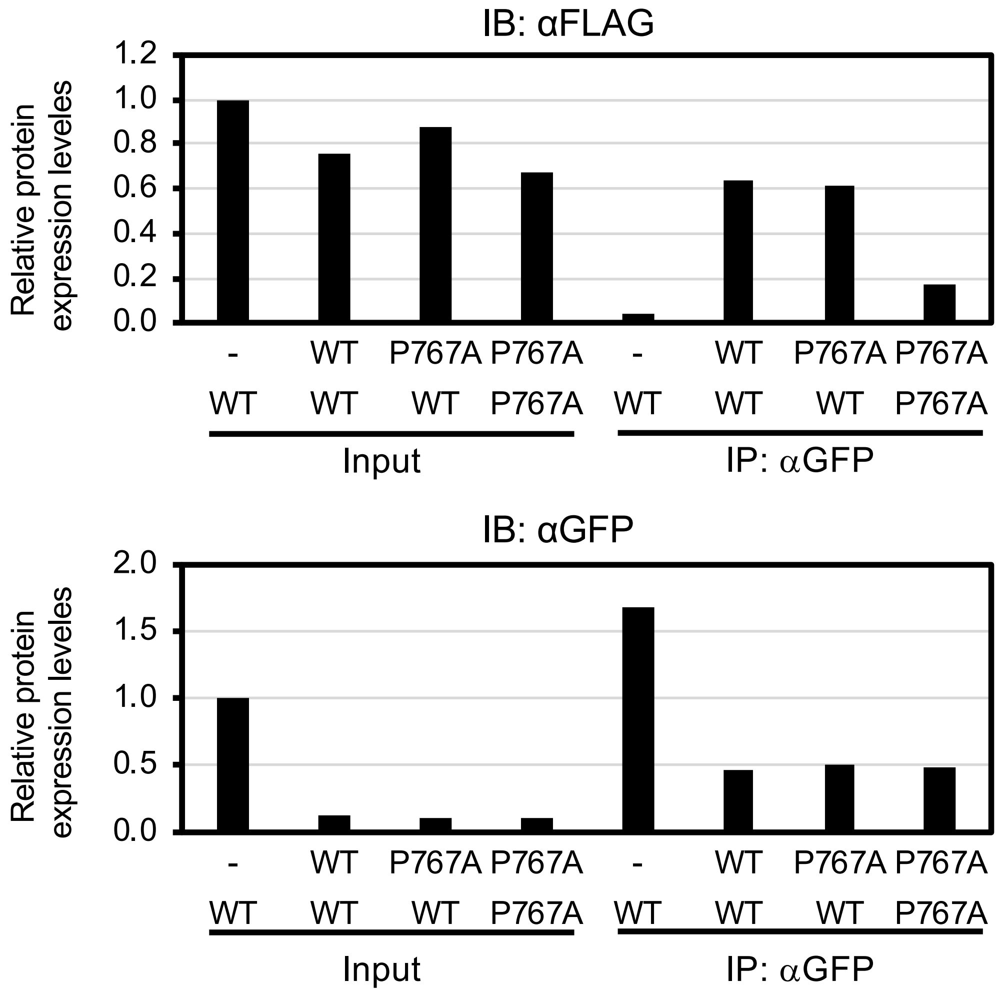

Supplemental Fig. 7

C

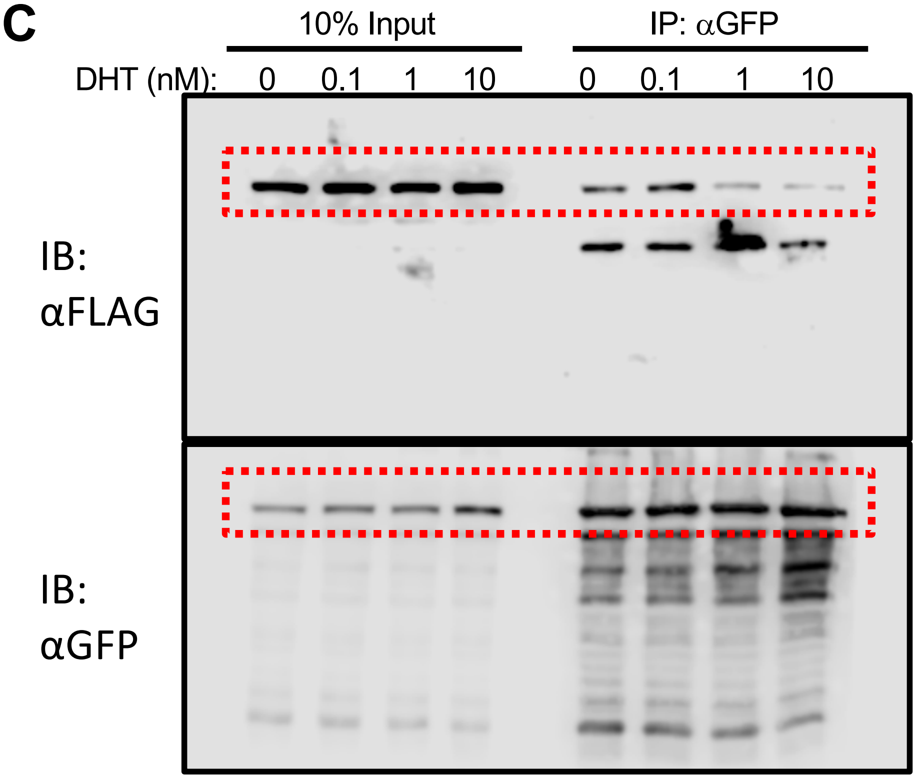

D

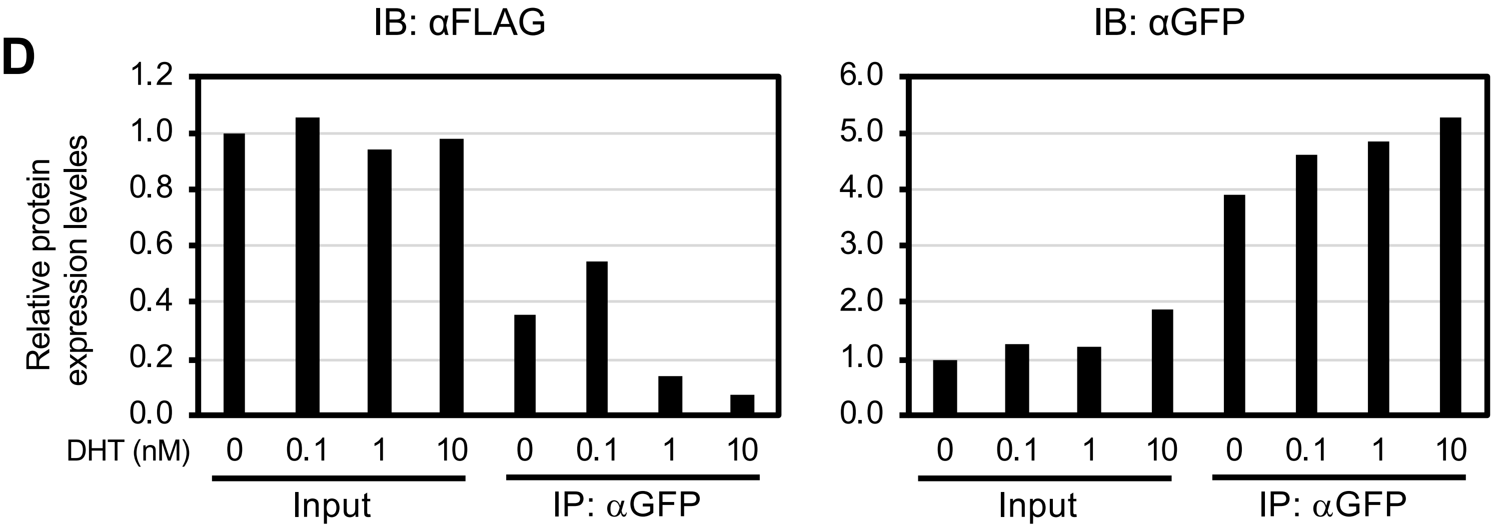

Supplemental Fig. 8

A

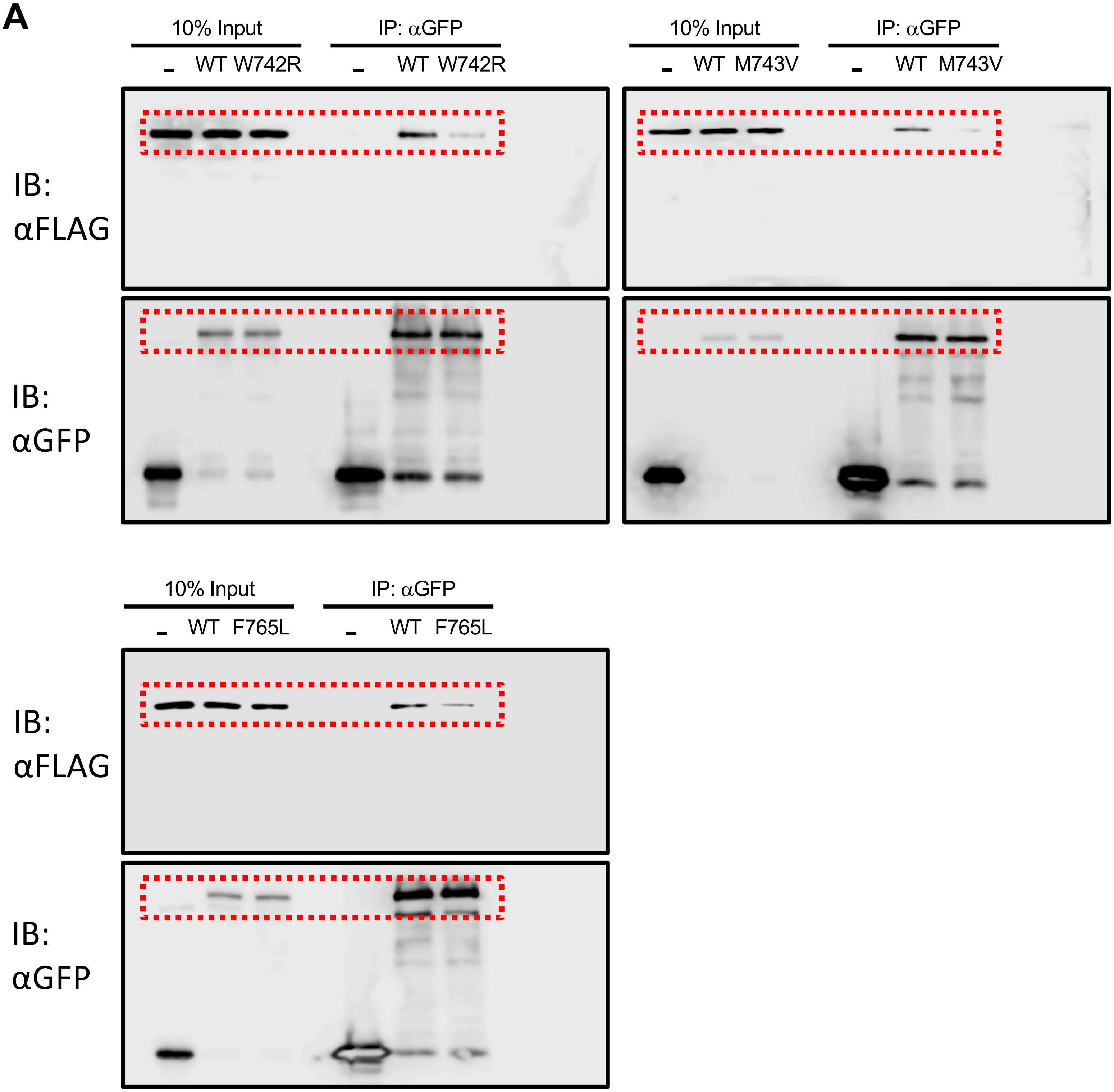

Supplemental Fig. 8

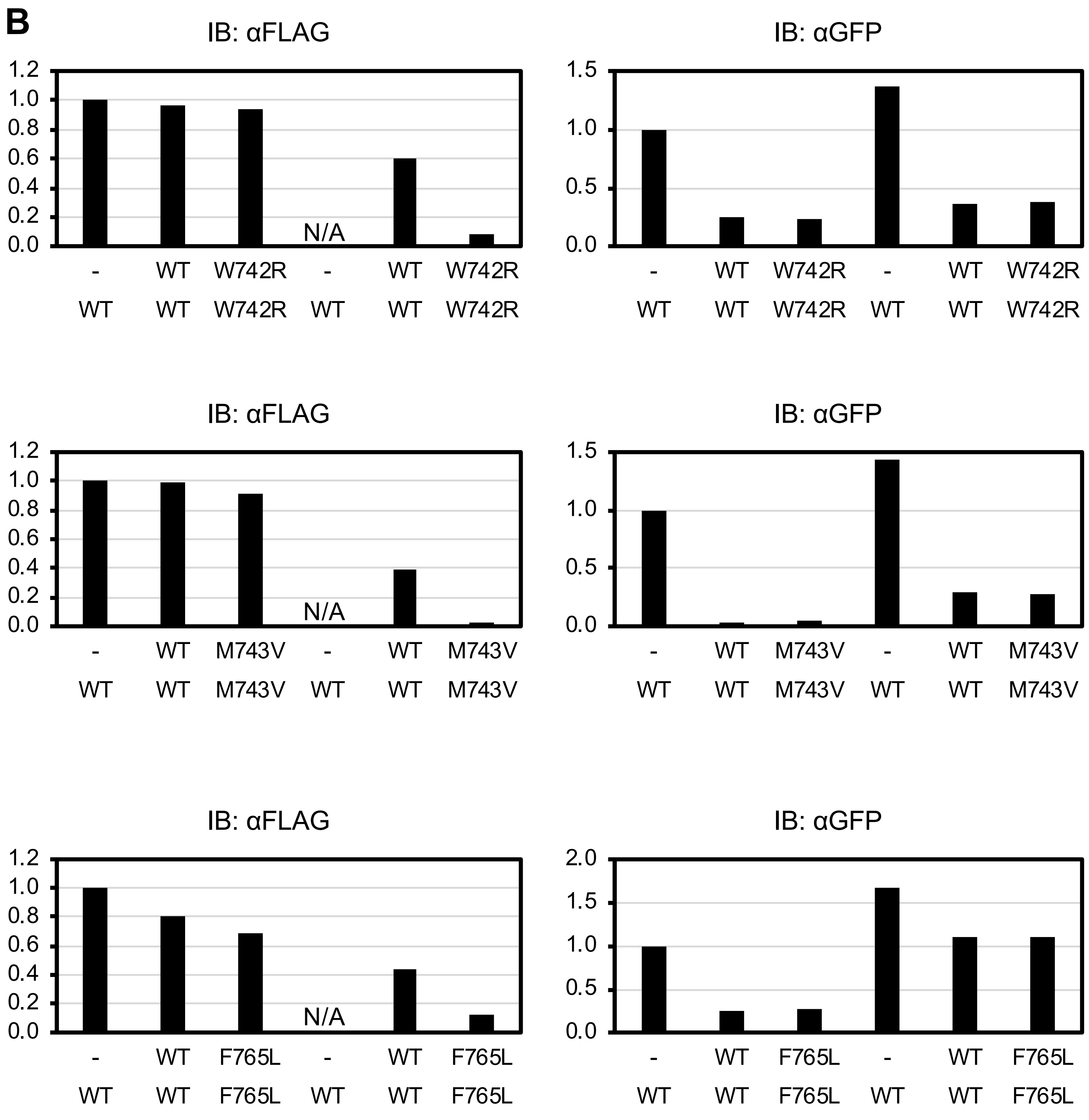

Supplement: Supplementary file 1 — Supplemental Figure1-8 [file 41598_2019_53139_MOESM1_ESM.pdf]
