# Supplementary figures and images for: Ligand induced dissociation of the AR homodimer precedes AR monomer translocation to the nucleus
Source: Sci Rep. 2019 Nov 13;9:16734. doi: 10.1038/s41598-019-53139-9 (PMC6853983; doi:10.1038/s41598-019-53139-9)

## Slide 1
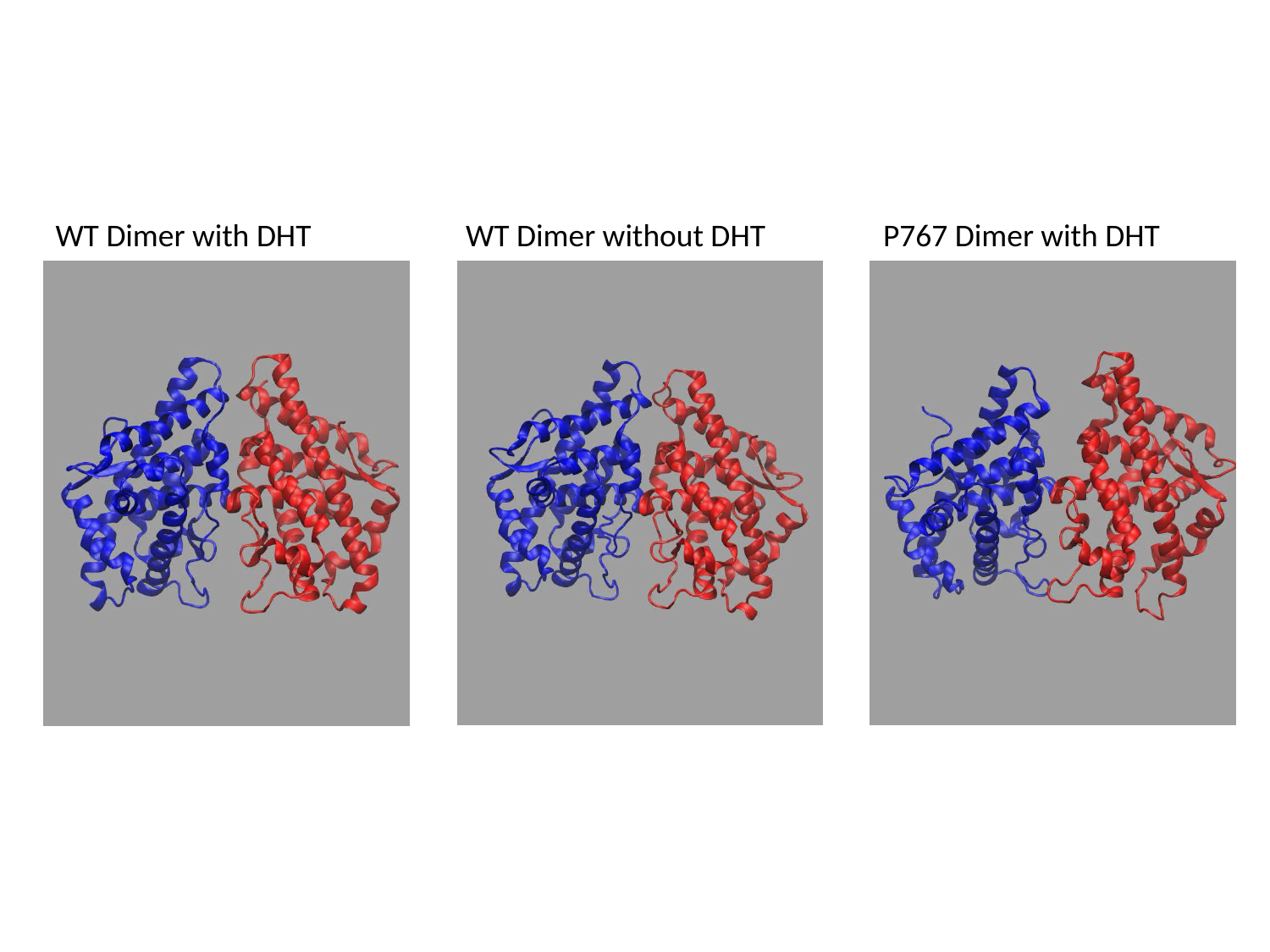

WT Dimer with DHT
WT Dimer without DHT
P767 Dimer with DHT

Supplement: Supplementary file 2 — Supplemental movie [file 41598_2019_53139_MOESM2_ESM.zip › supplemental movie.pptx]
